# Proteomic Analysis of *Oesophagostomum dentatum* (Nematoda) during Larval Transition, and the Effects of Hydrolase Inhibitors on Development

**DOI:** 10.1371/journal.pone.0063955

**Published:** 2013-05-22

**Authors:** Martina Ondrovics, Katja Silbermayr, Makedonka Mitreva, Neil D. Young, Ebrahim Razzazi-Fazeli, Robin B. Gasser, Anja Joachim

**Affiliations:** 1 Department of Pathobiology, Institute of Parasitology, University of Veterinary Medicine Vienna, Vienna, Austria; 2 Genome Institute, Washington University School of Medicine, St. Louis, Missouri, United States of America; 3 Division of Infectious Diseases, Department of Internal Medicine, Washington University School of Medicine, St. Louis, Missouri, United States of America; 4 Faculty of Veterinary Science, The University of Melbourne, Parkville, Victoria, Australia; 5 VetCore Facility for Research, University of Veterinary Medicine Vienna, Vienna, Austria; Griffith University, Australia

## Abstract

In this study, *in vitro* drug testing was combined with proteomic and bioinformatic analyses to identify and characterize proteins involved in larval development of *Oesophagostomum dentatum*, an economically important parasitic nematode. Four hydrolase inhibitors ο-phenanthroline, sodium fluoride, iodoacetamide and 1,2-epoxy-3-(*p*nitrophenoxy)-propane (EPNP) significantly inhibited (≥90%) larval development. Comparison of the proteomic profiles of the development-inhibited larvae with those of uninhibited control larvae using two-dimensional gel electrophoresis, and subsequent MALDI-TOF mass spectrometric analysis identified a down-regulation of 12 proteins inferred to be involved in various larval developmental processes, including post-embryonic development and growth. Furthermore, three proteins (i.e. intermediate filament protein B, tropomyosin and peptidyl-prolyl *cis-trans* isomerase) inferred to be involved in the moulting process were down-regulated in moulting- and development-inhibited *O. dentatum* larvae. This first proteomic map of *O. dentatum* larvae provides insights in the protein profile of larval development in this parasitic nematode, and significantly improves our understanding of the fundamental biology of its development. The results and the approach used might assist in developing new interventions against parasitic nematodes by blocking or disrupting their key biological pathways.

## Introduction

Parasitic roundworms (nematodes) of animals and humans are of major socioeconomic importance worldwide [1]–[5]. Of these nematodes, the soil-transmitted helminths (STHs) *Ancylostoma duodenale*, *Necator americanus*, *Trichuris trichiura* and *Ascaris* spp. are estimated to infect almost one sixth of the global human population [6], [7]. Also parasites of livestock, including species of *Haemonchus*, *Ostertagia*, *Teladorsagia*, *Trichostrongylus* and *Oesophagostomum*, collectively cause substantial economic losses estimated at billions of dollars per annum, due to poor productivity, failure to thrive, deaths and the cost of anthelmintic treatment [8]–[11]. In addition to their socioeconomic impact, widespread resistance in nematodes of livestock against the main classes of anthelmintics [12]–[14] has stimulated research toward designing alternative intervention and control strategies against these parasites. Central to this effort should be the discovery of new drug targets through an improved understanding of the fundamental biology of parasite development.

Despite the advances in ‘-omics’ and computer-based technologies [15], and extensive studies of the free-living nematode *Caenorhabditis elegans* [16]–[18], there is a paucity of information on developmental processes in parasitic nematodes of animals, particularly those of the order Strongylida, which are of major socioeconomic importance. Numerous studies (reviewed in [19]) show that the porcine nodule worm, *Oesophagostomum dentatum*, is a unique model for studying fundamental developmental and reproductive processes in strongylid nematodes because of its short life cycle and, particularly, an ability to maintain worms *in vitro* for weeks through multiple moults.

The life cycle of *O. dentatum* is simple and direct [20]. Unembryonated eggs are released in host faeces and develop into free-living, first- and second-stage larvae (L1s and L2s, respectively). Feeding on nutrients and microbes in the faecal matter, they develop into the infective, third-stage larvae (L3s) which are protected within a cuticular sheath. These larvae migrate from the faeces into the surrounding environment (pasture or soil), where the porcine host ingests them. Once ingested, the L3s exsheath in the small intestines of the pig *en route* to the large intestine. Upon reaching the large intestine, they burrow into the mucosal layer of the intestinal wall and subsequently produce lesions. Within the submucosa, the L3s moult to fourth-stage larvae (L4s) [21] and evoke an immune response that results in the encapsulation of the larvae in raised nodular lesions, made up mainly of aggregates of neutrophils and eosinophils [22]. Following the transition to the L4s, the larvae emerge from the mucosa within 6–17 days. The parasite undergoes another cuticular moult, subsequently maturing to an adult. The pre-patent period of *O. dentatum* is ∼17–20 days [23], although longer periods have been observed [20].

Recent transcriptomic studies [15], [24] have provided first insights into the molecular biology of different developmental stages of *O. dentatum*, leading to the characterization of a range of structural and functional molecules. In some studies of nematodes [25]–[31], various hydrolases (including cysteine, metallo-, serine and aspartic proteases, and pyrophosphatases) have been identified as key molecules likely to play essential and specific roles in parasite development, cuticle collagen processing and/or moulting processes and thus represent potential drug targets for nematocides. Having available a practical *in vitro* culture system for *O. dentatum* [32], [33] provides a unique opportunity to assess the effects of specific and selective inhibitors on protein expression in this parasite. In the present study, we selected inhibitors of the most relevant hydrolase groups involved in the development and moulting of parasitic nematodes [25]–[27], [30], [31], [34], [35], based on their ability to inhibit these processes without affecting viability and motility. We investigated the effects of these hydrolase inhibitors on the (phenotypic) proteomic profile of *O. dentatum* during its transition from the L3 to L4 stage using an integrated two-dimensional gel electrophoretic, mass spectrometric and bioinformatic approach, taking advantage of all of the currently available transcriptomic datasets for this parasitic nematode.

## Materials and Methods

### Ethics Statement

Experiments were conducted in accordance with the Austrian Animal Welfare Regulations and approved (permit GZ 68.205/103-II/10b/2008) by the Animal Ethics Committee of the University of Veterinary Medicine Vienna and the Ministry of Science.

### Parasite Material

A monospecific strain (OD-Hann) of *O. dentatum* was maintained routinely in experimentally infected pigs at the Institute of Parasitology, University of Veterinary Medicine Vienna. The faeces were collected to harvest L3s from coprocultures [23] and stored in distilled water at 11°C for a maximum of six months.

### Larval Development Inhibition Assay

The effects of seven different hydrolase inhibitors (Table 1) on larval development were assessed; the inhibitors included ο-phenanthroline monohydrate (1,10-phenanthroline; Carl Roth, Karlsruhe, Germany), a metalloprotease inhibitor; sodium fluoride (Merck, Darmstadt, Germany), a pyrophosphatase inhibitor; iodoacetamide (Sigma-Aldrich, St. Louis, USA), a cysteine protease inhibitor; 1,2-epoxy-3-(*p*nitrophenoxy)-propane (EPNP; Acros Organics, Geel, Belgium) and pepstatin A (Sigma-Aldrich), two aspartic protease inhibitors; and 4-(2-Aminoethyl) benzenesulfonyl fluoride (AEBSF; Roche Applied Science, Basel, Switzerland) and aprotinin (Sigma-Aldrich), two serine protease inhibitors. Ensheathed L3s were purified using a small-scale agar gel migration technique [23], [36] and then exsheathed in 12% (v/v) hypochlorite at 22°C [23]. The exsheated L3s were maintained in culture in 24-well plates (100 L3s/well) containing 1 ml of cultivation medium containing LB broth and 10% pig serum [37]. Plates were incubated at 38.5°C and 10% CO_2_ for 14 days, with a change of medium on days 4 and 11. To determine the effect of the different inhibitors on the development and moulting of the L3 to L4 stage of *O. dentatum*, the seven inhibitors were added (individually) to replicate cultures at ascending concentrations (ο-phenanthroline 3.125 µM–2 mM; sodium fluoride 0.5 mM–10 mM; iodoacetamide 5 µM–500 mM; EPNP 100 µM–4 mM; pepstatin A 0.05 µM–175 µM; AEBSF 250 µM–1 mM; aprotinin 0.77 µM–1.16 mM; see Table 1). The percentage of developed L4s was determined on days 4, 7, 10 and 14. Phenotypes (viability, motility and mortality) were recorded at 100x magnification using an inverted light microscope (Diaphot 300, Nikon Corporation). Each inhibitor and each control were run in triplicate, and the respective solvent controls were included in each assay.

**Table 1:** List of the hydrolase inhibitors tested.

Inhibitor	Inhibited hydrolase class	CAS-no.	Tested concentrations	Optimal concentration forinhibition	CHEMBL Compound ID
o-phenanthroline	Metalloprotease	5144-89-8	3.125 µM–2 mM	12.5 µM	415879
Sodium fluoride	Pyrophosphatase	7681-49-4	0.5 mM–10 mM	5 mM	1528
Iodoacetamide	Cysteine protease	144-48-9	5 µM–500 mM	125 µM	276727
EPNP^a^	Aspartic protease	5255-75-4	100 µM–4 mM	1.4 mM	33775
Pepstatin A	Aspartic protease	26305-03-3	0.05 µM–175 µM	/	296588
AEBSF	Serine protease	34284-75-8	250 µM–1 mM	/	1256178
Aprotinin	Serine protease	9087-70-1	0.77 µM–1.16 mM	/	1201619

a EPNP, 1,2 epoxy-3-(pnitrophenoxy)-propane.

To test for the reversibility of inhibition of development, the larvae were first cultured for seven days in medium containing inhibitors at concentrations with a known inhibitory effect of ≥90%. Then, the medium was replaced with inhibitor-free medium following several washes, and the larvae maintained until day 14.

### Protein Extraction

L3s of *O. dentatum* (*n* = 500,000), cultured *in vitro* for four days with or without the effective hydrolase inhibitors, were harvested, washed three times in phosphate-buffered saline (PBS; pH 7.4), snap frozen in liquid nitrogen and ground to fine powder with mortar and pestle pre-frozen in liquid nitrogen. Proteins were resuspended in ice-cold 10% (v/v) TCA in acetone at −20°C and precipitated for 90 min. After precipitation, proteins were centrifuged at 4°C at 17,500 *g* for 15 min. The supernatant was discarded, and the pellet washed twice with chilled (−20°C) 100% acetone and centrifuged to remove any traces of TCA. Finally, acetone was removed by evaporation at 22°C. Proteins were resuspended overnight in 250–500 µl solubilisation buffer [7 M urea, 2 M thiourea, 4% (w/v) 3-[(3-cholamidopropyl)dimethylammonio]-2-hydroxy-1-propanesulfonate (CHAPS; Carl Roth) and 30 mM Tris-Base (Carl Roth)] at 22°C. Insoluble material was removed by centrifugation at 241,800 *g* at 20°C for 30 min. The supernatant was collected and the total protein content of each sample determined [38] using bovine serum albumin (BSA) as a standard.

### Two-dimensional Electrophoresis

For separation in the first dimension, an aliquot of 120 µg of parasite protein was diluted in a final volume of 300 µl of rehydration solution [8 M urea, 2% (w/v) CHAPS, 12.7 mM dithiothreitol (DTT), 2% immobilized pH gradient (IPG) buffer 3–10 non-linear (GE Healthcare Life Sciences, Freiburg, Germany)] and used to rehydrate 13 cm IPG strips with a non-linear gradient pH 3–10 (Immobiline, GE Healthcare Life Sciences) for 18 h at 22–24°C. Isoelectric focusing (IEF) was carried out (300 V ascending to 3,500 V for 90 min, followed by 3,500 V for 18 h) using a Multiphor II electrophoresis chamber (GE Healthcare Life Sciences). After IEF, the IPG strips were incubated at 22°C for 20 min in an equilibration buffer (6 M urea, 2% (w/v) sodium dodecyl-sulphate (SDS), 30% (v/v) glycerol, 150 mM Tris-HCl, pH 8.8, 64 mM DTT). A second equilibration was performed in the same buffer, except that DTT was replaced by 135 mM iodoacetamide. The IPG strips were then washed with deionized water. In the second dimension, SDS-PAGE was performed in vertical slab gels (1.5 mm; T = 12%, C = 2.6%, 1.5 M Tris-HCl, pH 8.8, 10% SDS) under reducing conditions at 15 mA for 15 min, followed by 25 mA in a Protean II electrophoresis chamber (Bio-Rad Laboratories, Hercules, USA). Gels were stained with silver [39]. Two technical replicates and one biological replicate were run for each treatment. Gels were scanned using the program ImageMaster™ 2D platinum v.7.0 (GE Healthcare Life Sciences). Proteins that were significantly (*p*≤0.05) differentially expressed were selected for further analyses by mass spectrometry combined with bioinformatics.

### Sample Preparation for Mass Spectrometric Analysis

Protein spots were excised manually from silver-stained two-dimensional electrophoretic (2-DE) gels. ‘Negative’ control spots were also excised from blank regions of each gel and processed in parallel. Spots were washed, reduced with DTT and alkylated with iodoacetamide. In-gel digestion with trypsin (Trypsin Gold, Mass Spectrometry Grade, Promega, Madison, USA) was carried out [40]. In order to enhance peptide ionisation and the sensitivity of mass spectrometry, dried peptides were de-salted using Zip-Tips µC_18_ (Millipore, Billerica, USA) according to the manufacturer’s instructions.

### Spotting and Mass Spectrometry

Peptides were annotated using a Matrix Assisted Laser Desorption Ionisation Tandem Time-of-Flight (MALDI-TOF/TOF) mass spectrometer (Ultraflex II; Bruker Daltonics, Bremen, Germany) in MS and MS/MS modes. De-salted peptides (0.5 µl) were spotted on to a disposable AnchorChip MALDI target plate pre-spotted with α-cyano-4-hydroxycinnamic acid (PAC target; Bruker Daltonics). MS spectral data were acquired from the samples and a MS/MS list was generated for further analysis based on the most intense ions present (trypsin and major keratin ions were excluded). Spectra processing and peak annotation were carried out using FlexAnalysis and Biotools (Bruker Daltonics).

### Data Analysis

All peptide mass fingerprint (PMF) data were refined using MS/MS data. Protein sequence matches with a significant MS/MS score (*p*<0.05) were used for further analysis. Ion mass data and amino acid sequences characterized from processed spectra were compared with data in public eukaryotic databases, including an inferred protein database of *O. dentatum* (www.nematode.net), the UniProt database (http://www.uniprot.org/) and the National Center for Biotechnology Information (NCBI) non-redundant (nr) database (http://www.ncbi.nlm.nih.gov) using the following search parameters: global modifications carbamido-methylation on cysteine; variable modifications oxidation on methionine; peptide mass tolerance = 100 ppm; MS/MS tolerance = 1 Da; one missed cleavage allowed. The programs Proteinscape (v.2.1; Bruker Daltonics) and MASCOT (www.matrixscience.com) were used for data management and analyses. Proteins identified by MS were annotated based on their closest homologue (BLASTp, *E*-value cut-off ≤1×10^−5^) in the UniProt database and Gene Ontology (GO). Subsequent GO analyses were performed using the AmiGO BLAST tool [41]. Protein sequences were also mapped to known biological pathways (KOBAS algorithm) [42] using BLASTp (*E*-value cut-off ≤10^−5^) based on sequence homology to molecules in the Kyoto Encyclopedia of Genes and Genomes (KEGG) database [43].

### Statistical Analysis

Statistical calculations were performed using SPSS (v.20.0) for windows. To test for significant differences in the percentage of L4 development, the development-inhibited groups and the controls were compared using the Kruskal-Wallis test and Mann-Whitney *U*-test.

## Results

### Testing of Inhibitors

**Figure 1:**
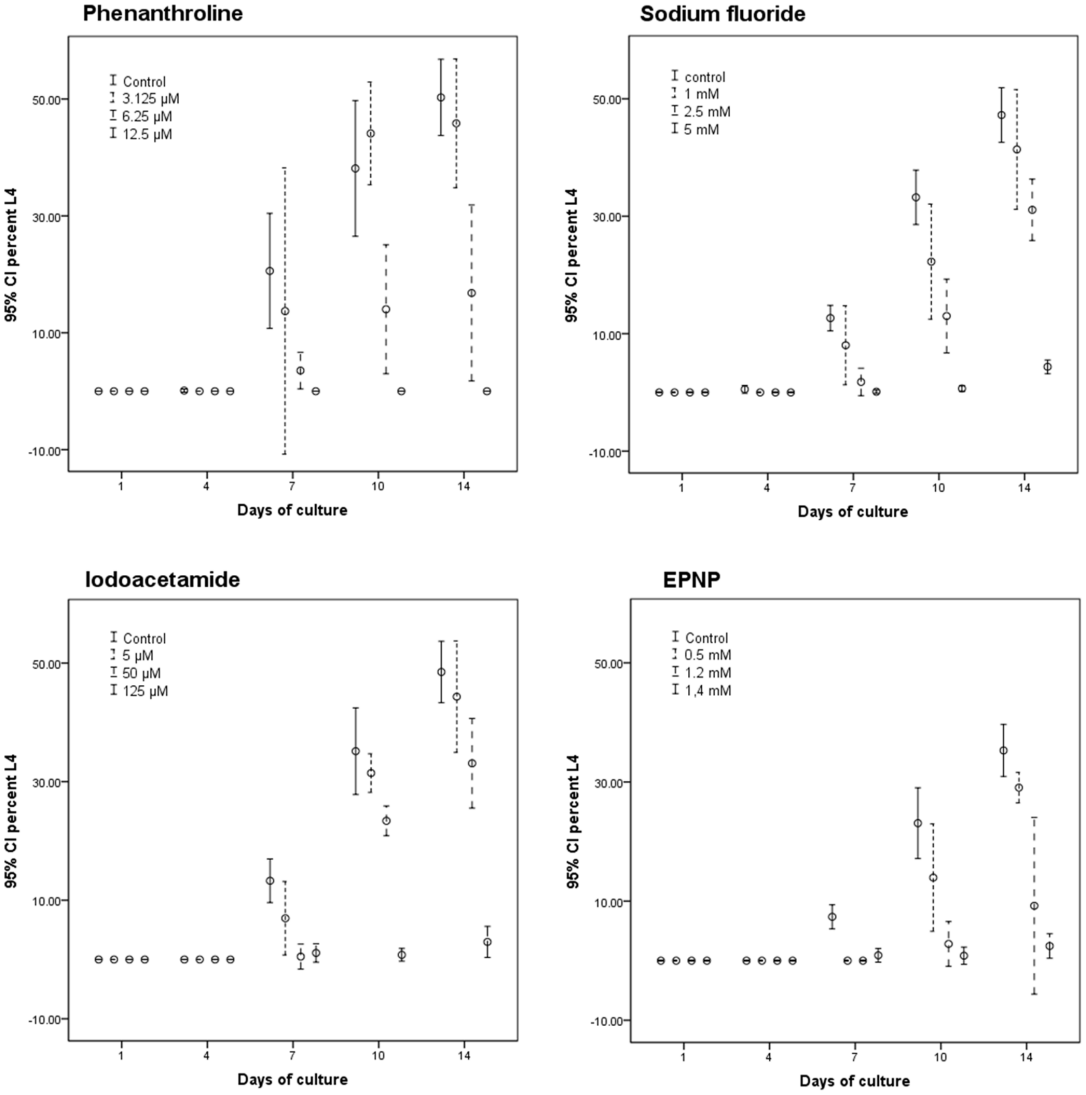
Error bar plot of the tested hydrolase inhibitors with a significant inhibitory effect on larval development of *O. dentatum* larvae cultured *in vitro*. *O. dentatum* larvae were cultured *in vitro* for 14 days with or without enzyme inhibitors, and the percentage of L3s that developed to L4s was determined on days 4, 7, 10 and 14. The addition of four different enzyme inhibitors (o-phenanthroline, sodium fluoride, iodoacetamide and EPNP) to cultivation media resulted in a significant (*p*≤0.01) developmental inhibition of *O. dentatum* larvae when compared with untreated controls.

We assessed the inhibitory effects of each compound on the development from the exsheathed L3 to the L4 stage of *O. dentatum* (Figure 1) by culturing larvae for 14 days in medium containing seven different hydrolase inhibitors (Table 1). A significant inhibitory effect (*p*≤0.01) was detected for four of the seven hydrolase inhibitors tested. By adding iodoacetamide (125 µM), the development of L3s to L4s was inhibited by 93.9±5.1% compared with untreated controls. The addition of 1.4 mM EPNP resulted in 93.0±5.6% inhibition. Complete inhibition of nematode development was achieved by adding 12.5 µM o-phenanthroline to the *in vitro* cultures. Sodium fluoride (5 mM) resulted in a 90.8±3.2% inhibition. No significant inhibitory effect was observed for the aspartic protease inhibitor pepstatin A or either serine protease inhibitors (AEBSF and aprotinin) tested (data not shown). The addition of higher concentrations of each of the four inhibitory compounds did not result in an increased inhibitory effect, but did lead to a higher mortality of larvae. The reversibility of the inhibitory effect of each inhibitor was assessed. The removal of iodoacetamide, EPNP, o-phenanthroline and sodium fluoride resulted in 90.5%, 85.6%, 89.5%, and 106.3%, respectively, of L3s developing through to L4s with respect to the controls.

### Two-dimensional Gel Electrophoretic Analysis

We explored the induced changes in the *O. dentatum* larval proteome, following treatment with inhibitors and associated inhibition of development. For this purpose, protein extracts were prepared from whole worms for 2-DE; the average recovery was 7.4 µg protein per 1,000 larvae. The results of 2-DE showed that the protein expression profile varied between the development-inhibited and control cultures. Protein spots were distributed throughout the whole pH range (3–10), but more spots were in the neutral and acidic pH ranges. The proteins differentially expressed between the development-inhibited and the control larvae were displayed and then annotated.

### Annotation of Proteins

**Figure 2:**
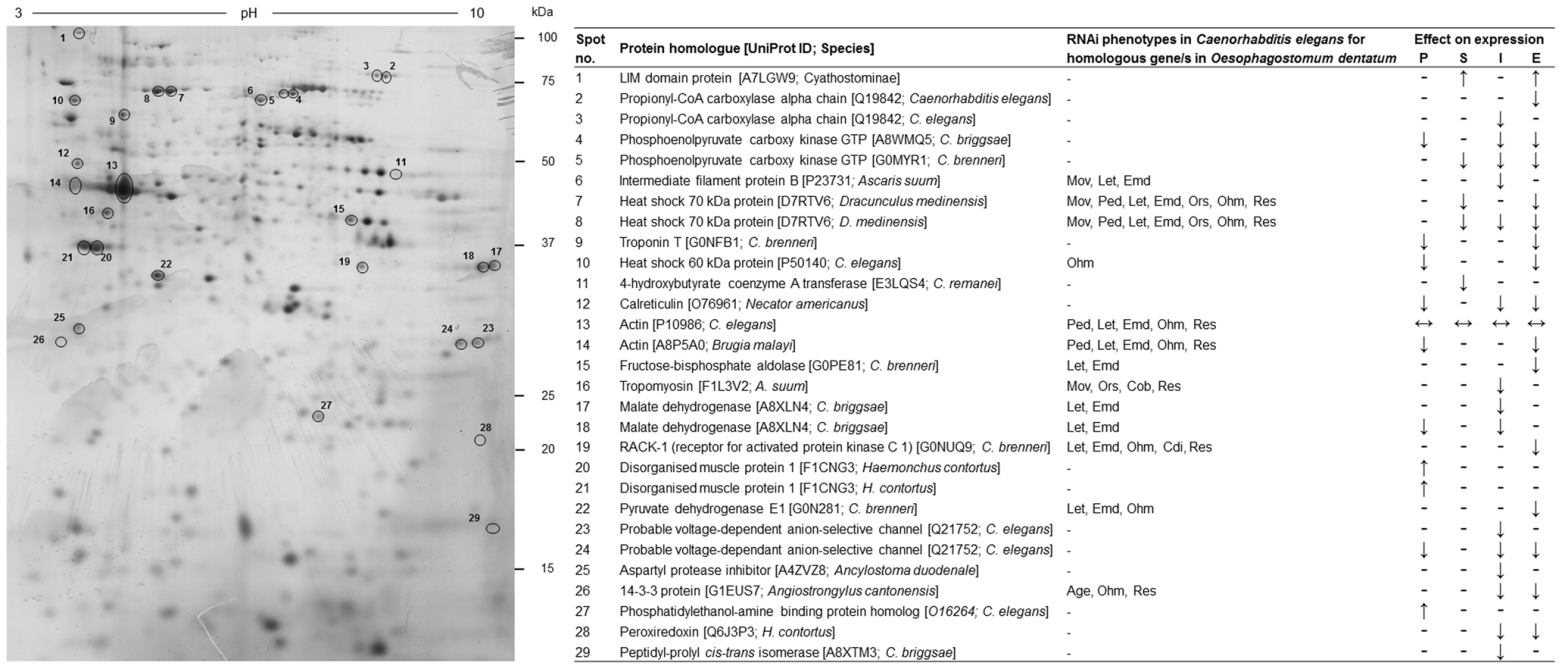
Two-dimensional electrophoretic gel of *O. dentatum* L3s displaying protein spots, non wild-type RNAi phenotypes in *Caenorhabditis elegans* linked to gene homologues in *O. dentatum* and the effect of inhibitors (i.e. P, o-phenanthroline; S, sodium fluoride; I, iodoacetamide; E, 1,2 epoxy-3-(*p*nitrophenoxy)-propane) on protein expression in development-inhibited larvae (↓, down-regulation; ↑, up-regulation; ↔, no difference; -, not studied). Significantly (*p*≤0.05) differentially expressed protein spots selected for mass spectrometric analyses are numbered. Applying 2-DE analysis followed by MALDI-TOF-MS/MS resulted in the identification of 29 spots representing 22 different proteins. Protein homologues were identified in the UniProt database and are indicated [UniProt ID; Species]. RNAi phenotypes were linked to 13 of 29 homologues in *C. elegans*: Mov, Movement variant; Let, Lethal; Emd, Embryonic development variant; Ped, Postembryonic development variant; Ors, Organism segment morphology variant; Ohm, Organism homeostasis metabolism variant; Res, Reproductive system physiology variant; Cob, Cell organization biogenesis variant; Cdi, Cell division variant; Age, Life span variant.

The significantly (*p*≤0.05) differentially expressed spots were excised from the 2-DE gels, digested with trypsin and then analysed by MALDI-TOF-MS/MS. The most intense spot (no. 13) representing an actin isoform was present on all gels, was constitutively expressed and was thus selected as reference spot. This approach allowed us to reproducibly resolve 29 spots representing 22 different proteins (Figure 2, Tables 2 and 3), of which 21 (except spots 2 and 3) shared sequence homology to individual conceptually translated proteins of *O. dentatum*. Spots 2 and 3 had significant amino acid sequence homology to propionyl-CoA carboxylase alpha-chain of *C. elegans* (Table 2). Both spots independently yielded a significant MASCOT MS/MS score of 166.4 (spot 2) and 148.5 (spot 3), inferring that these results are highly reliable.

**Table 2:** Annotation of the different protein spots inferred for *O. dentatum* and MALDI-TOF-MS/MS results.

Spot no.	Contig ID	MASCOT Score (MS/MS)	%Cov	#Pep	Theoretical pI/MW	Protein homologue	MASCOT Score (MS/MS)	%Cov	#Pep
1	Oden_isotig07502	90.8	13.1	2	4.9/20.1	/	/	/	/
2	/	/	/	/	8.5/79.7	Propionyl-CoA carboxylase alpha chain [*Caenorhabditis elegans*]	166.4	4.4	2
3	/	/	/	/	8.5/79.7	Propionyl-CoA carboxylase alpha chain [*C. elegans*]	148.5	4.4	2
4	Oden_isotig22486	166.1	15	3	5.8/20.1	Phosphoenolpyruvate carboxy kinase GTP [*Ascaris suum*]	136.1	10.2	2
5	Oden_isotig22486	111.7	15	3	5.8/20.1	Phosphoenolpyruvate carboxy kinase GTP [*A. suum*]	86.0	10.2	2
6	Oden_isotig18493	306.1	8.4	4	5.8/66.5	Intermediate filament protein [*A. suum*]	306.1	8.3	4
7	Oden_isotig01423	370	9.3	4	5.5/61.2	Heat shock protein 70 [*Angiostrongylus vasorum*]	370.0	10.1	4
8	Oden_isotig01423	320.2	9.3	2	5.5/61.2	Heat shock protein 70 *[A. vasorum*]	320.2	10.1	3
9	Oden_isotig19560	94.4	13.8	3	9.1/37.8	/	/	/	/
10	Oden_isotig13569	132.6	8.7	3	5.0/53.2	/	/	/	/
11	Oden_isotig13083	144.2	18.2	3	6.9/42.0	Hypothetical protein CRE_26209 [*C. remanei*]	100.4	5.9	2
12	Oden_isotig17754	314.9	22.5	6	4.6/37.1	Calreticulin [*Necator americanus*]	133.3	7.4	2
13	Oden_isotig20090	289.1	14	3	5.3/36.1	/	/	/	/
14	Oden_isotig19833	126.8	9.9	2	5.2/35.2	/	/	/	/
15	Oden_isotig21929	147	14.1	3	9.0/20.6	/	/	/	/
16	Oden_isotig07123	121.9	24.4	4	4.5/23.9	/	/	/	/
17	Oden_isotig07234	387.3	19.9	3	9.4/34.8	/	/	/	/
18	Oden_isotig07234	197.1	12.2	2	9.4/34.8	/	/	/	/
19	Oden_isotig20226	249.1	21.1	3	6.6/37.3	/	/	/	/
20	Oden_isotig20183	498.5	24.2	5	5.1/35.8	Disorganised muscle protein 1 [*Haemonchus contortus*]	498.5	24.3	5
21	Oden_isotig20183	510.8	24.2	5	5.1/35.8	Disorganised muscle protein 1 [*H. contortus*]	510.8	24.3	5
22	Oden_isotig12108	260.8	15.3	4	5.8/38.3	/	/	/	/
23	Oden_isotig14559	225.1	19.3	2	9.4/30.0	/	/	/	/
24	Oden_isotig14559	154.9	12.1	2	9.4/30.0	/	/	/	/
25	Oden_isotig21295	99.9	17.3	3	5.0/25.2	/	/	/	/
26	Oden_isotig20385	120	18.6	3	5.1/26.0	/	/	/	/
27	Oden_isotig11085	155.1	15	2	7.2/20.6	/	/	/	/
28	Oden_isotig11077	248.9	19.2	3	6.3/21.6	/	/	/	/
29	Oden_isotig21414	127.7	14.6	2	8.4/18.5	/	/	/	/

%Cov, percentage sequence coverage. #Pep, number of peptides found within the sequence for the resulting inferred protein.

**Table 3:** Protein homologues for the different spots.

Spot no.	Contig ID	Protein identity	Protein homologue	Species	UniProt ID	Score	Identity [%]	*E*-value
1	Oden_isotig07502	LIM domain protein	LIM domain protein variant	Cyathostominae	A7LGW9	741	83	9*10^−77^
2	/	Propionyl-CoA carboxylase alpha chain	/	*Caenorhabditis elegans*	Q19842	/	/	/
3	/	Propionyl-CoA carboxylase alpha chain	/	*C. elegans*	Q19842	/	/	/
4+5	Oden_isotig22486	Phosphoenolpyruvate carboxy kinase GTP	Putative uncharacterized protein	*C. briggsae*	A8WMQ5	787	80	4*10^−82^
4+5	Oden_isotig23105	Phosphoenolpyruvate carboxy kinase GTP	Putative uncharacterized protein	*C. brenneri*	G0MYR1	937	83	3*10^−99^
6	Oden_isotig18493	Intermediate filament protein B	Intermediate filament protein B	*Ascaris suum*	P23731	2715	91	0
7+8	Oden_isotig01423	Heat shock 70 kDa protein	Heat shock protein 70	*Dracunculus medinensis*	D7RTV6	2771	92	0
9	Oden_isotig19560	Troponin T	CBN-TNT-2 protein	*C. brenneri*	G0NFB1	1392	83	1*10^−151^
10	Oden_isotig13569	Heat shock 60 kDa protein	Chaperonin homolog Hsp-60, mitochondrial	*C. elegans*	P50140	2086	83	0
11	Oden_isotig13083	4-hydroxybutyrate coenzyme A transferase	Putative uncharacterized protein	*C. remanei*	E3LQS4	1738	87	0
12	Oden_isotig17754	Calreticulin	Calreticulin	*Necator americanus*	O76961	1700	93	0
13	Oden_isotig20090	Actin	Actin-4	*C. elegans*	P10986	1670	100	0
14	Oden_isotig19833	Actin	Actin, putative	*Brugia malayi*	A8P5A0	1629	99	1*10^−179^
15	Oden_isotig21929	Fructose-bisphosphate aldolase	Fructose-bisphosphate aldolase	*C. brenneri*	G0PE81	834	86	2*10^−87^
16	Oden_isotig07123	Tropomyosin	Tropomyosin	*A. suum*	F1L3V2	908	91	6*10^−96^
17+18	Oden_isotig07234	Malate dehydrogenase	Malate dehydrogenase	*C. briggsae*	A8XLN4	1370	82	1*10^−149^
19	Oden_isotig20226	RACK-1 (receptor for activated protein kinase C 1)	CBN-RACK-1 protein	*C. brenneri*	G0NUQ9	1492	84	1*10^−163^
20+21	Oden_isotig20183	Disorganised muscle protein 1	Disorganised muscle protein 1	*Haemonchus contortus*	F1CNG3	1675	98	0
22	Oden_isotig12108	Pyruvate dehydrogenase E1	Putative uncharacterized protein	*C. brenneri*	G0N281	1464	79	1*10^−160^
23+24	Oden_isotig14559	Probable voltage-dependent anion-selective channel	Probable voltage-dependent anion-selective channel	*C. elegans*	Q21752	1236	81	1*10^−133^
25	Oden_isotig21295	Aspartyl protease inhibitor	Aspartyl protease inhibitor 1	*Ancylostoma duodenale*	A4ZVZ8	948	80	1*10^−100^
26	Oden_isotig20385	14-3-3 protein	14-3-3 protein	*Angiostrongylus cantonensis*	G1EUS7	1130	99	1*10^−121^
27	Oden_isotig11085	Phosphatidylethanol-amine binding protein homolog	Phosphatidylethanolamine-binding protein homolog F40A3.3	*C. elegans*	O16264	731	71	2*10^−75^
28	Oden_isotig11077	Peroxiredoxin	Peroxiredoxin	*H. contortus*	Q6J3P3	924	88	7*10^−98^
29	Oden_isotig21414	Peptidyl-prolyl *cis-trans* isomerase	Peptidyl-prolyl *cis-trans* isomerase	*C. briggsae*	A8XTM3	867	92	2*10^−91^

### Homology-based Search

The amino acid sequences predicted from contigs (Table 2) were searched against nematode molecules in the UniProt database. At least one nematode homologue was identified for each *O. dentatum* sequence using an *E*-value cut-off of ≤2×10^−50^. UniProt BLAST scores of sequence similarity for each sequence (for the five best matches) ranged from 731 to 2771. Homologous proteins were identified in other strongylid species (e.g., species of Cyathostominae, *Haemonchus contortus*, *Angiostrongylus cantonensis*, *A. duodenale* and/or *N. americanus*) as well as in related orders, such as the Rhabditida (e.g., *Caenorhabditis* spp.), Spirurida (e.g., *Brugia malayi* and/or *Dracunculus medinensis*) and Ascaridida (e.g., *Ascaris suum*) (Table 2; Table S1). These results confirmed correct spectra assignments for individual spots (nos. 2–8, 11, 12, 20 and 21) with significant MASCOT MS/MS scores for homologues in other species of nematodes (cf. Tables 2 and 3). All assigned contigs had homologous proteins in *C. elegans*. Thirteen of 29 protein spots (44.8%) were linked to distinct ‘non-wildtype’ double-stranded RNA interference (RNAi) phenotypes (Figure 2; Table S2) recorded in at least two different experiments (cf. WormBase): lethality (*n* = 10), defects in embryonic development (*n* = 10), organism homeostasis metabolism (*n* = 8), physiology of the reproductive system (*n* = 7), movement (*n* = 4) and post-embryonic development (*n* = 4). In *C. elegans*, no ‘non-wildtype’ RNAi phenotypes for *O. dentatum* homologues of the LIM domain protein, propionyl-CoA carboxylase, phosphoenolpyruvate carboxy kinase GTP, troponin T, 4-hydroxybutyrate coenzyme A transferase, calreticulin, disorganised muscle protein 1, probable voltage-dependant anion-selective channel, aspartyl protease inhibitor, phosphatidylethanol-amine binding protein, peroxiredoxin and peptidyl-prolyl *cis-trans* isomerase have yet been reported in more than two different experiments (cf. WormBase).

### KEGG Analysis

To further categorize key differentially expressed proteins and identify specific pathways in which these proteins are predicted to be involved, homologues to curated proteins in the KEGG database were identified (see Tables S3 and S4). In depth analysis revealed ‘cellular processes’, ‘genetic information processing’ and ‘metabolism’ as the three main protein classification terms (Table S3A). The class ‘cellular processes’, including the subclass ‘cytoskeleton proteins’ comprised the structural protein actin (spots 13 and 14). The KEGG classification ‘genetic information processing’ (including several subclasses) was linked to four proteins (representing 18.2% of all proteins), namely heat shock 70 kDa protein (HSP-70; spots 7 and 8), heat shock 60 kDa protein (HSP-60; spot 10), calreticulin (spot 12) and actin (spots 13 and 14). Seven proteins (representing 31.8% of all proteins) could be assigned to the class ‘metabolism’ and represented enzymes. Six of the seven enzymes [namely propionyl-CoA carboxylase (EC 6.4.1.3; spots 2 and 3), phosphoenolpyruvate carboxy kinase GTP (EC 4.1.1.32; spots 4 and 5), fructose-bisphosphate aldolase (EC 4.1.2.13; spot 15), malate dehydrogenase (EC 1.1.1.31; spots 17 and 18), pyruvate dehydrogenase E1 (EC 1.2.4.1; spot 22) and peptidyl-prolyl *cis-trans* isomerase (EC 5.2.1.8; spot 29)] are involved in various pathways linked to amino acid, carbohydrate and/or energy metabolism.

The KEGG pathway classification included the biological pathway terms ‘cellular processes’ (*n* = 4), ‘environmental information processing’ (*n* = 2), ‘genetic information processing’ (*n* = 3), ‘metabolism’ (*n* = 5) and ‘organismal systems’ (*n* = 5) (Table S3B). No annotation was found in the KEGG database for nine proteins (representing 36.4% of all proteins), including LIM domain protein (spot 1), intermediate filament protein B (spot 6), troponin T (spot 9), 4-hydroxybutyrate coenzyme A transferase (spot 11), tropomyosin (spot 16), receptor for activated protein kinase C 1 (RACK-1, spot 19), disorganised muscle protein 1 (DIM-1; spots 20 and 21), aspartyl protease inhibitor (spot 25) and phosphatidylethanol-amine binding protein (spot 27).

### Gene Ontology (GO) Analysis

In order to better understand the biological processes and molecular functions in which the identified proteins are inferred to be involved, GO analyses were performed using the AmiGO BLAST tool. Of the 22 proteins, 19 (86.4%) could be assigned to GO classifications associated with biological processes and 18 proteins (81.8%) were assigned to certain molecular functions. Only GO annotations found in related nematode species (particularly *C. elegans*) were taken into account using a cut-off of *p*≤2×10^−25^ (see Tables S5 and S6). GO classification analysis revealed associations of the proteins identified to a range of biological processes (Table S5A), including ‘reproduction’ (*n* = 8), ‘metabolic process’ (*n* = 10), ‘cellular process’ (*n* = 9), ‘multicellular organismal growth’ (*n* = 11), ‘developmental process’ (*n* = 11), ‘growth’ (*n* = 11), ‘locomotion’ (*n* = 8), ‘response to stimulus’ (*n* = 8), ‘localization’ (*n* = 4) and ‘biological regulation’ (*n* = 8). Most proteins assigned to the GO term ‘metabolic process’ related to ‘cellular metabolic process’ (representing 70% of the enzymes identified). No GO classifications for biological processes were found for three of the 22 proteins, namely DIM-1 (spots 20 and 21), aspartyl protease inhibitor (spot 25) and phosphatidylethanol-amine binding protein (spot 27). ‘binding’ (*n* = 11) and ‘catalytic activity’ (*n* = 10) were the two main functional GO categories (Table S5B); additional GO categories included ‘structural molecule activity’ (*n* = 2), ‘transporter activity’ (*n* = 1) and ‘antioxidant activity’ (*n* = 1). In depth analysis revealed that the majority (63.6%) of proteins assigned to ‘binding’ are involved in ‘protein binding’ processes. No GO annotation for molecular functions was found for troponin T (spot 9), DIM-1 (spots 20 and 21), aspartyl protease inhibitor (spot 25) or phosphatidylethanol-amine binding protein (spot 27).

## Discussion

Drug resistance represents a major concern in the control of parasitic nematodes [12]–[14], [44]. Therefore, much research is directed towards the development of new agents in the treatment of nematode infections. Proteins involved in fundamental developmental processes in nematodes represent promising targets for the design of new and selective interventions. Hence, this study aimed at identifying and characterizing proteins involved in the larval development of *O. dentatum*, a model organism representative of parasitic nematodes of major socioeconomic impact. We applied an integrative approach combining *in vitro* drug testing with proteomic and bioinformatic analyses to provide first insights into larval development in *O. dentatum*.

To generate a development-inhibited phenotype for *O. dentatum*, hydrolase inhibitors were selected based on their ability to impede the moulting and development of larvae without affecting their viability and motility. Enzyme inhibitors were tested to cover the most relevant hydrolase classes known to be involved in the highly sophisticated moulting process in various nematode species [25]–[27], [30], [31], [34], [35]. Significant inhibition of moulting and development has been described previously in a range of nematodes and could be confirmed in *O. dentatum* for the three inhibitors, ο-phenanthroline [27], [34], [45], sodium fluoride [25], [26] and iodoacetamide [28]. The inhibitory effect of EPNP had not been tested before on the development of nematode larvae. In malaria parasites, however, this protease inhibitor disintegrates gametocyte membranes [46], and it has been used frequently as an inhibitor of peptidases of the A1 family [47]. This is particularly interesting, since the other aspartic protease inhibitor tested, pepstatin A, was neither effective in our model nor in related nematodes [27], [34], probably due to its limited half-life.

Since the development of nematode larvae and the sophisticated moulting process require a series of structural, biochemical, metabolic and physiological changes, we assumed that the proteins essential for fundamental developmental processes in development-inhibited larvae are less abundantly expressed compared with those of uninhibited controls in which normal development occurred. Thus, protein spots that were significantly differentially expressed between development-inhibited and control larvae were subjected to mass spectrometric and bioinformatic analyses. Using this approach, we identified 29 spots representing 22 different proteins. Several spots located at different positions in the same gel were inferred to be distinct protein isoforms or the same protein with varying post-translational modifications. Furthermore, 27 out of 29 identified spots were encoded in *O. dentatum* contigs derived from ESTs generated by 454 sequencing (FLX GS20 sequencer) [48] and analyzed using an advanced bioinformatic pipeline [49] for the interference of key biological processes [50], such as development and moulting. The *O. dentatum* contigs were further subjected to homology-based functional annotation. All *O. dentatum* amino acid sequences characterised had homologous proteins in other nematodes, including species from the orders Strongylida, Rhabditida, Spirurida, and Ascaridida.

The proteins identified and annotated included structural and cytoskeletal proteins (intermediate filament protein B, troponin T, actin, tropomyosin, DIM-1), enzymes involved in metabolism (propionyl-CoA carboxylase, phosphoenolpyruvate carboxy kinase GTP, 4-hydroxybutyrate coenzyme A transferase, fructose-bisphosphate aldolase, malate dehydrogenase, pyruvate dehydrogenase E1, ‘probable voltage-dependent anion-selective channel’ and peptidyl-prolyl *cis-trans* isomerase), stress-associated peptides (HSP-60, HSP-70, calreticulin and peroxiredoxin), regulatory and interacting peptides (LIM domain protein, RACK-1, 14-3-3 protein and phosphatidylethanol-amine binding protein) and one protease-inhibiting protein (aspartyl protease inhibitor). The expression of the vast majority (*n* = 19) of the 22 proteins identified was down-regulated in the development-inhibited group compared with controls, whereas three proteins (DIM-1, LIM domain protein and phosphatidylethanol-amine binding protein) were shown to be up-regulated. We functionally annotated 19 (86.4%) of 22 protein sequences using GO and established pathway associations for 14 (63.6%) of 22 sequences in KEGG. The annotation of the 22 proteins revealed specific roles in larval developmental processes for intermediate filament protein B, HSP-70, troponin T, HSP-60, calreticulin, actin, fructose-bisphosphate aldolase, tropomyosin, malate dehydrogenase, RACK-1, pyruvate dehydrogenase E1 and 14-3-3 protein. The expression of all 12 proteins was down-regulated in the development-inhibited larvae compared with controls. This finding indicates important roles for these proteins in nematode development.

KEGG analysis identified seven proteins (propionyl-CoA carboxylase, phosphoenolpyruvate carboxy kinase GTP, fructose-bisphosphate aldolase, malate dehydrogenase, pyruvate dehydrogenase E1, peroxiredoxin and peptidyl-prolyl *cis-trans* isomerase) as enzymes involved in amino acid, carbohydrate and/or energy metabolic pathways. Phosphoenolpyruvate carboxy kinase GTP, fructose-bisphosphate aldolase, malate dehydrogenase and pyruvate dehydrogenase E1 are known to be involved in the glucose associated energy metabolic pathways in nematodes [51]–[54], whereas propionyl-CoA carboxylase is involved in fatty acid synthesis [55]. The ‘probable voltage-dependent anion-selective channel’ protein likely plays pivotal roles in the parasite’s metabolisms, since it is involved in calcium signalling pathways and ion transport [56]. Peroxiredoxins are a ubiquitous family of antioxidant proteins and contribute to the oxidative-stress response of multicellular organisms. In nematodes, antioxidant enzymes and stress-associated proteins are central to the protection against oxygen radicals [57]. The expression of all enzymes and all stress-associated proteins identified in our study was down-regulated in development-inhibited larvae compared with larvae exhibiting physiological moulting and growth patterns. We hypothesize that the moulting process is accompanied by increased energy consumption as well as augmented metabolic and physiological activities. These changes were particularly evident when the protein profiles of larvae with physiological developmental patterns were compared with those whose moulting had been inhibited with one of the abovementioned hydrolase inhibitors, respectively.

Intermediate filament protein B, actin, troponin T, tropomyosin and DIM-1 are structural proteins ubiquitously expressed in all eukaryotic cells. They play essential roles in myofibril assembly and muscle contraction [58]–[60], and provide structure and stability [61]–[63]. In *C. elegans* (wild type strain of variation Bristol N2), the silencing of the gene encoding for tropomyosin, *lev-11*, results in embryonic lethality and worms that are paralyzed and showed abnormal muscle filament assembly [64], [65]. The over-expression of DIM-1 in the development-inhibited larvae might be a response to the reduced expression of the other structural proteins identified. Thus, DIM-1 could be part of a regulatory system and might be over-expressed to ensure stability and structure in *O. dentatum* larvae. The multifaceted and crucial functions of the abovementioned proteins indicate their importance in fundamental developmental processes. Interestingly, the two structural proteins, namely intermediate filament protein B and tropomyosin are known to play pivotal roles in the moulting process of *C. elegans* by remodelling the attachments between the muscle, the hypodermis and the exoskeleton [16], [18]. Additionally, the protein peptidyl-prolyl *cis-trans* isomerase is proposed to be involved in the moulting process in nematodes [66]. Besides their function as molecular chaperones and their involvement in stress responses and cell signalling [67]–[69], peptidyl-prolyl *cis-trans* isomerases of *C. elegans* are responsible for proper collagen biosynthesis [66] during the moulting process. These proteins represent a large multi-gene family which has also been identified in *B. malayi* as well as in other parasitic and free-living nematodes, including *Onchocerca volvulus*, *H. contortus*, *Caenorhabditis briggsae* and *C. elegans* [67], [70]–[72]. Interestingly, we observed a down-regulation of expression of the proteins intermediate filament protein B, tropomyosin and peptidyl-prolyl *cis-trans* isomerase, all homologues of which are involved in the moulting process in *C. elegans*. The moulting- and development-inhibited larvae expressed these three proteins to a lesser extent compared with the control larvae, which exhibited normal development and need the involvement of the moulting-associated proteins to accomplish the initiation and completion of their moulting. These three proteins, that are known to be involved in the moulting process, represent particularly promising candidate drug targets against parasitic nematodes, since they are essential for one of the most determining parts in the parasite’s development, appear to be conserved across nematode species and are not present in the mammalian host (pig).

Two proteins, LIM domain protein and phosphatidylethanol-amine binding protein (PEBP), are associated with ion and lipid binding, respectively. LIM domain proteins are essential in a variety of fundamental biological processes [73] by mediating protein-protein interactions [74]. PEBPs are highly conserved and function in lipid binding, serine protease inhibition [75] and the regulation of several signalling pathways, such as the MAP kinase [76] and the NF-kappaB [77] pathways. Members of the PEBP family include the *Ov*-16 antigen of *O. volvulus* [78] and the excretory-secretory antigen 26 of *Toxocara canis* [79]. In *B. malayi*, a homologue of this protein is highly abundant in excretory/secretory products [72], [80]. Since both of these proteins were over-expressed in the development-inhibited *O. dentatum*, we hypothesize that the addition of the hydrolase inhibitors might lead to an up-regulation of specific interactions and signalling pathways, for reasons that remain to be elucidated. These modified processes might be associated with an increased involvement of LIM domain protein and phosphatidylethanol-amine binding protein, in which they might fulfil various protein interaction and/or pathway regulatory functions.

### Conclusion

The findings of this study support the hypothesis that, in development-inhibited larvae of *O. dentatum*, proteins essential for developmental processes are less abundantly expressed compared with the uninhibited controls. Twelve proteins putatively involved in various larval developmental processes as well as three proteins involved in the moulting process were identified to be down-regulated in the moulting- and development-inhibited *O. dentatum* larvae. A better understanding of such key developmental processes paves the way to new interventions against parasitic nematodes by blocking or disrupting key biological pathways in them. Thus, this study provides a foundation on which future research on fundamental developmental processes in parasitic nematodes could be built. Furthermore, the present findings enable us to focus on specific proteins involved in the moulting cascade in *O. dentatum*. In future studies, the protein expression of exsheathed, ensheathed and moulting *O. dentatum* larvae could be compared using DIGE technologies [81] and supported by real-time PCR analysis of transcription. We aim to identify and characterize proteins involved in the moulting process and assess their merit as drug targets. The discovery of new drug targets is of particular importance, considering the increasing prevalence and distribution of drug resistance in parasitic nematodes of major socioeconomic importance.

## Supporting Information

Table S1 **Detailed list of protein homologues including the five best matches for each protein identification.** The table lists the the ID number of the *Oesophagostomum dentatum* contig (Contig ID) and the individual protein identities (Protein identity), the five highest scoring putative proteins identified in homology-based search using the UniProt database (Protein homologue), the species in which these proteins occur (Species), the UniProt accession number of these proteins (UniProt ID), the score for sequence similarity (Score), the per cent of sequence identity (Identitiy [%]) and the expectation value (*E*-value).
(PDF)

Table S2 **RNAi phenotypes in** ***Caenorhabditis elegans*** **for homologous gene/s in** ***Oesophagostomum dentatum*** **.**
(PDF)

Table S3 **A, B. KEGG pathway analysis of the proteins identified.**
(PDF)

Table S4 **Detailed list of the protein assignments to KEGG BRITE protein families and to KEGG biological pathways.**
(PDF)

Table S5 **A, B. Gene Ontology (GO) analysis of the proteins identified.**
(PDF)

Table S6 **Gene Ontology (GO) assigments of proteins identified.** The conceptually translated amino acid sequences of the identified *Oesophagostomum dentatum* contigs were applied to GO analyses using the AmiGO tool. Only annotations found in related nematode species were taken into account using a cut-off of *p*≤2×10^−25^.
(PDF)
