# Supplementary material for: Proteomic Analysis of Oesophagostomum dentatum (Nematoda) during Larval Transition, and the Effects of Hydrolase Inhibitors on Development
Source: PLoS One. 2013 May 22;8(5):e63955. doi: 10.1371/journal.pone.0063955 (PMC3661580; doi:10.1371/journal.pone.0063955)
Supplement: Table S1 — Detailed list of protein homologues including the five best matches for each protein identification. The table lists the the ID number of the Oesophagostomum dentatum contig (Contig ID) and the individual protein identities (Protein identity), the five highest scoring putative proteins identified in homology-based search using the UniProt database (Protein homologue), the species in which these proteins occur (Species), the UniProt accession number of these proteins (UniProt ID), the score for sequence similarity (Score), the per cent of sequence identity (Identitiy [%]) and the expectation value (E-value). (PDF) [file pone.0063955.s001.pdf]

Table S1. Detailed list of protein homologues including the five best matches for each protein identification. The table lists the the ID number of the *Oesophagostomum dentatum* contig (Contig ID) and the individual protein identities (Protein identity), the five highest scoring putative proteins identified in homology-based search using the UniProt database (Protein homologue), the species in which these proteins occur (Species), the UniProt accession number of these proteins (UniProt ID), the score for sequence similarity (Score), the per cent of sequence identity (Identitiy [%]) and the expectation value (*E*-value).

Spot no.	Contig ID	Protein identity	Protein homologue	Species	UniProt ID	Score	Identity [%]	*E*-value
1	Oden_isotig07502	LIM domain protein	LIM domain protein variant	Cyathostominae	A7LGW9	741	83	$9*10^{-77}$
			CBN-TAG-273 protein	*Caenorhabditis brenneri*	G0N6V2	527	63	$6*10^{-52}$
			Putative uncharacterized protein	*C. remanei*	E3N762	522	63	$2*10^{-51}$
			CBR-TAG273 protein	*C. briggsae*	A8Y0E8	522	63	$2*10^{-51}$
			Protein TAG-273, isoform b	*C. elegans*	Q86CZ7	518	62	$7*10^{-51}$
2		Propionyl-CoA carboxylase alpha chain		*C. elegans*	Q19842			
3		Propionyl-CoA carboxylase alpha chain		*C. elegans*	Q19842			
4 + 5	Oden_isotig22486	Phosphoenolpyruvate carboxy kinase GTP	Putative uncharacterized protein	*C. briggsae*	A8WMQ5	787	80	$4*10^{-82}$
			Putative uncharacterized protein	*C. brenneri*	G0MYR1	779	80	$4*10^{-81}$
			Protein W05G11.6	*C. elegans*	Q8ITY2	774	79	$1*10^{-80}$
			Putative uncharacterized protein	*C. remanei*	E3MSQ2	751	73	$6*10^{-78}$
			Phosphoenolpyruvate carboxykinase [GTP]	*Ascaris suum*	Q05893	746	75	$2*10^{-77}$
	Oden_isotig23105	Phosphoenolpyruvate carboxy kinase GTP	Putative uncharacterized protein	*C. brenneri*	G0MYR1	937	83	$3*10^{-99}$
			Putative uncharacterized protein	*C. briggsae*	A8WMQ5	926	81	$5*10^{-98}$
			Putative uncharacterized protein	*C. remanei*	E3MSQ2	920	81	$2*10^{-97}$
			Protein W05G11.6	*C. elegans*	Q8ITY2	906	80	$1*10^{-95}$
			Phosphoenolpyruvat carboxylase [GTP]	*A. suum*	Q05893	884	82	$4*10^{-93}$
6	Oden_isotig18493	Intermediate filament protein B	Intermediate filament protein B	*A. suum*	P23731	2715	91	0
			CRE-IFB-1 protein	*C. remanei*	E3M4V6	2686	90	0
			Intermediate filament protein ifb-1	*C. elegans*	Q19289	2681	90	0
			Putative uncharacterized protein	*C. brenneri*	G0NAS5	2681	90	0
			CBR-IFB-1 protein	*C. briggsae*	A8WU67	2542	91	0
7 + 8	Oden_isotig01423	Heat shock 70 kDa protein	Heat shock protein 70	*Dracunculus medinensis*	D7RTV6	2771	92	0
			Putative uncharacterized protein	*Loa loa*	E1FQI9	2757	92	0
			Heat shock 70 kDa protein, putative	*Brugia malayi*	A8Q5Z6	2751	91	0
			CBN-HSP-1 protein	*C. brenneri*	G0MRH9	2750	92	0
			Heat shock 70 kDa protein A	*C. elegans*	P09446	2749	92	0
9	Oden_isotig19560	Troponin T	CBN-TNT-2 protein	*C. brenneri*	G0NFB1	1392	83	$1*10^{-151}$
			CBR-TNT-2 protein	*C. briggsae*	A8XMD1	1378	82	$1*10^{-150}$
			Cre-TNT-2 protein	*C. remanei*	E3MUG5	1358	81	$1*10^{-147}$
			Protein TNT-2, isoform a	*C. elegans*	Q7Z072	1352	80	$1*10^{-147}$
			CRE-MUP-2 protein	*C. remanei*	E3LDI9	1301	79	$1*10^{-147}$
10	Oden_isotig13569	Heat shock 60 kDa protein	Chaperonin homolog Hsp-60, mitochondrial	*C. elegans*	P50140	2086	83	0
			CBR-HSP-60 protein	*C. briggsae*	A8XDX6	2086	82	0
			CBN-HSP-60 protein	*C. brenneri*	G0NBT5	2084	82	0
			CRE-HSP-60 protein	*C. remanei*	E3MNJ4	2073	82	0
			Chaperonin-like protein HSP60	*B. malayi*	D0VBH6	2065	81	0
11	Oden_isotig13083	4-hydroxybutyrate coenzyme A transferase	Putative uncharacterized protein	*C. remanei*	E3LQS4	1738	87	0
			Putative uncharacterized protein	*C. briggsae*	A8XCM4	1707	85	0
			Putative uncharacterized protein	*C. brenneri*	G0PJF7	1706	85	0
			Protein C44B7.10	*C. elegans*	Q18599	1690	84	0
			4-hydroxybutyrate coenzyme A transferase	*A. suum*	F1KWR3	1601	79	$1*10^{-176}$
12	Oden_isotig17754	Calreticulin	Calreticulin	*Necator americanus*	O76961	1700	93	0
			Calreticulin	*Heligmosomoides polygyrus*	Q0VJ74	1683	91	0
			Calreticulin	*Angiostrongylus cantonensis*	G0YXZ1	1678	91	0
			Calreticulin-like protein	*Haemonchus contortus*	Q6R5P2	1670	94	0
			Calreticulin	*A. suum*	F1L1U2	1596	86	$1*10^{-175}$
13	Oden_isotig20090	Actin	Actin-4	*C. elegans*	P10986	1670	100	0
			Actin	*C. remanei*	Q4TTR3	1670	100	0
			Putative uncharacterized protein	*C. brenneri*	G0N8H7	1670	100	0
			Actin variant 2	*Dictyocaulus viviparus*	B0LJD2	1670	100	0
			Actin-2	*A. suum*	F1L3U5	1663	99	0
14	Oden_isotig19833	Actin	Actin, putative	*B. malayi*	A8P5A0	1629	99	$1*10^{-179}$
			Putative uncharacterized protein	*L. loa*	E1GDP0	1629	99	$1*10^{-179}$
			Actin	*Wuchereria bancrofti*	Q9NJ12	1629	99	$1*10^{-179}$
			Actin	*A. suum*	F1LAW8	1623	99	$1*10^{-179}$
			Actin-2	*C. elegans*	P10984	1608	97	$1*10^{-176}$

15	Oden_isotig21929	Fructose-bisphosphate aldolase	Fructose-bisphosphate aldolase	*C. brenneri*	G0PE81	834	86	$2*10^{-87}$
			Fructose-bisphosphate aldolase	*C. briggsae*	A8X7W5	833	86	$3*10^{-87}$
			Fructose-bisphosphate aldolase	*C. remanei*	E3LYT0	832	86	$3*10^{-87}$
			Fructose-bisphosphate aldolase 2	*C. elegans*	P46563	822	84	$5*10^{-86}$
			Fructose-bisphosphate aldolase	*Heterodera glycines*	Q9GPK1	737	74	$4*10^{-76}$
16	Oden_isotig07123	Tropomyosin	Tropomyosin	*A. suum*	F1L3V2	908	91	$6*10^{-96}$
			Isoform d of Tropomyosin isoforms a/b/d/f	*C. elegans*	Q22866-2	901	90	$4*10^{-95}$
			Tropomyosin	*Teladorsagia circumcinta*	D2Y180	883	90	$5*10^{-93}$
			Tropomyosin	*H. polygyrus*	A8D2L1	883	90	$5*10^{-93}$
			Tropomyosin, muscle	*Trichostrongylus colubriformis*	P15846	873	89	$7*10^{-92}$
17 + 18	Oden_isotig07234	Malate dehydrogenase	Malate dehydrogenase	*C. briggsae*	A8XLN4	1370	82	$1*10^{-149}$
			Malate dehydrogenase	*C. brenneri*	G0MWK6	1368	81	$1*10^{-149}$
			Probable malate dehydrogenase, mitochondrial	*C. elegans*	O02640	1354	80	$1*10^{-147}$
			Malate dehydrogenase	*C. angaria*	B6VBX1	1345	80	$1*10^{-146}$
			Malate dehydrogenase	*C. remanei*	E3LX21	1336	78	$1*10^{-145}$
19	Oden_isotig20226	RACK-1 (receptor for activated protein kinase C 1)	CBN-RACK-1 protein	*C. brenneri*	G0NUQ9	1492	84	$1*10^{-163}$
			CBR-RACK-1 protein	*C. briggsae*	A8WUZ1	1491	84	$1*10^{-163}$
			CRE-RACK-1 protein	*C. remanei*	E3MM37	1489	84	$1*10^{-163}$
			Guanine nucleotide-binding protein	*C. elegans*	Q21215	1483	84	$1*10^{-162}$
			Activated protein kinase C receptor RACK1	*L. loa*	E1FVP8	1343	77	$1*10^{-146}$
20 + 21	Oden_isotig20183	Disorganised muscle protein1	Disorganised muscle protein 1	*H. contortus*	F1CNG3	1675	98	0
			Disorganised muscle protein 1, putative	*B. malayi*	A8Q0T1	1521	88	$1*10^{-166}$
			Disorganised muscle protein 1	*L. loa*	E1FM88	1520	88	$1*10^{-166}$
			As37	*A. suum*	Q8MY16	1519	87	$1*10^{-166}$
			Disorganised muscle protein 1	*C. elegans*	Q18066	1313	77	$1*10^{-142}$
22	Oden_isotig12108	Pyruvate Dehydrogenase E1	Putative uncharacterized protein	*C. brenneri*	G0N281	1464	79	$1*10^{-160}$
			Putative uncharacterized protein	*C. remanei*	E3NEV6	1442	77	$1*10^{-157}$
			Putative uncharacterized protein	*C. briggsae*	A8XZA8	1441	78	$1*10^{-157}$
			Pyruvate dehydrogenase E1 component subunit beta, mitochondrial	*C. elegans*	O44451	1436	78	$1*10^{-156}$
			Pyruvate dehydrogenase E1 component subunit beta	*A. suum*	F1L1Q5	1322	73	$1*10^{-143}$
23 + 24	Oden_isotig14559	Probable voltage-dependent anion-selective channel	Probable voltage-dependent anion-selective channel	*C. elegans*	Q21752	1236	81	$1*10^{-133}$
			Putative uncharacterized protein	*C. remanei*	E3M7D7	1227	80	$1*10^{-132}$
			Putative uncharacterized protein	*C. briggsae*	A8X1E2	1212	78	$1*10^{-131}$
			Putative uncharacterized protein	*C. brenneri*	G0NRV4	1208	78	$1*10^{-130}$
			Probable voltage-dependent anion-selective channel	*B. malayi*	A8PQQ0	789	51	$8*10^{-82}$
25	Oden_isotig21295	Aspartyl protease inhibitor	Aspartyl protease inhibitor 1	*Ancylostoma ceylanicum*	Q64FN5	948	81	$1*10^{-100}$
			Aspartyl protease inhibitor 1	*A. duodenale*	A4ZVZ8	948	80	$1*10^{-100}$
			Aspartyl protease inhibitor 1	*A. caninum*	Q64FN6	948	80	$1*10^{-100}$
			Aspartyl protease inhibitor 1	*T. columbiformis*	P59704	782	64	$3*10^{-81}$
			Aspartyl protease inhibitor 1	*O. ostertagi*	Q95PP1	758	61	$2*10^{-78}$
26	Oden_isotig20385	14-3-3 protein	14-3-3 protein	*A. cantonensis*	G1EUS7	1130	99	$1*10^{-121}$
			14-3-3 protein isoform 2	*A. caninum*	C3VXZ2	1122	98	$1*10^{-120}$
			14-3-3-like protein	*A. suum*	F1KXW6	120	97	$1*10^{-120}$
			14-3-3 protein	*Bursaphelenchus xylophilus*	D1MBS7	1102	95	$1*10^{-118}$
			14-3-3-like protein 2	*C. elegans*	Q20655	1098	96	$1*10^{-118}$
27	Oden_isotig11085	Phosphatidylethanol-amine binding protein homolog	Phosphatidylethanolamine-binding protein homolog F40A3.3	*C. elegans*	O16264	731	71	$2*10^{-75}$
			Putative uncharacterized protein	*C. remanei*	E3LK80	726	71	$3*10^{-75}$
			Putative uncharacterized protein	*C. brenneri*	G0MTP8	712	71	$2*10^{-73}$
			Phosphatidylethanolamine-binding protein	*A. suum*	F1L5C6	712	69	$3*10^{-73}$
			OV-16 antigen	*L. loa*	E1GEX5	702	68	$4*10^{-72}$
28	Oden_isotig11077	Peroxiredoxin	Peroxiredoxin	*H. contortus*	Q6J3P3	924	88	$7*10^{-98}$
			Thioredoxin peroxidase	*Ostertagia ostertagi*	Q8WQ02	891	87	$5*10^{-94}$
			Protein PRDX-2, isoform a	*C. elegans*	Q8IG31	876	84	$3*10^{-92}$
			Putative uncharacterized protein	*C. brenneri*	G0PDG1	871	83	$7*10^{-98}$
			CRE-PRDX-2 protein	*C. remanei*	E3M4K3	870	83	$1*10^{-91}$
29	Oden_isotig21414	Peptidyl-prolyl *cis-trans* isomerase	Peptidyl-prolyl *cis-trans* isomerase	*C. briggsae*	A8XTM3	867	92	$2*10^{-91}$
			Peptidyl-prolyl *cis-trans* isomerase 3	*C. elegans*	P52011	851	90	$2*10^{-89}$
			Peptidyl-prolyl *cis-trans* isomerase	*C. remanei*	E3LLL1	171	90	$5*10^{-88}$
			Peptidyl-prolyl *cis-trans* isomerase	*C. brenneri*	G0N4S5	826	88	$1*10^{-86}$
			Peptidyl-prolyl *cis-trans* isomerase	*L. loa*	E1FRU8	807	85	$2*10^{-84}$
