# Supplementary material for: Proteomic Analysis of Oesophagostomum dentatum (Nematoda) during Larval Transition, and the Effects of Hydrolase Inhibitors on Development
Source: PLoS One. 2013 May 22;8(5):e63955. doi: 10.1371/journal.pone.0063955 (PMC3661580; doi:10.1371/journal.pone.0063955)
Supplement: Table S2 — RNAi phenotypes in Caenorhabditis elegans for homologous gene/s in Oesophagostomum dentatum . (PDF) [file pone.0063955.s002.pdf]

Table S2. RNAi phenotypes in *Caenorhabditis elegans* for homologous gene/s in *Oesophagostomum dentatum*.

C. elegans **RNAi phenotypes**	**Spot no. [References]**
Behavior variant	
0001206 Movement variant (Mov)	6 [1,2], 7 [2-4], 8 [2-4], 16 [5-7]
Development variant	
0000049 Postembryonic development variant (Ped)	7 [2,8,9], 8 [2,8,9], 13 [4,10], 14 [4,10]
0000062 Lethal (Let)	6 [2,10-14], 7 [2,10,15], 8 [2,10,15], 13 [6,11,16], 14 [6,11,16], 15 [1,11], 17 [1,2,10,11,17], 18 [1,2,10,11,17], 19 [1,6,18,19], 22 [1,2,10,11]
0000749 Embryonic development variant (Emd)	6 [2,11-14], 7 [2,10,15], 8 [2,10,15], 13 [6,11,16], 14 [6,11,16], 15 [1,11], 17 [1,2,10,11,17], 18 [1,2,10,11,17], 19 [1,6,18,19], 22 [1,2,10,11]
Morphology variant	
0000582 Organism segment morphology variant (Ors)	7 [2,4], 8 [2,4], 16 [10,21]
0010002 Cell organization biogenesis variant (Cob)	16 [7,22]
Physiology variant	
0000039 Life span variant (Age)	26 [23-25]
0000577 Organism homeostasis metabolism variant (Ohm)	7 [9,26-28], 8 [9,26-28], 10 [17,29], 13 [28,30,31], 14 [28,30,31], 19 [1,2,18,20,32], 22 [1,2], 26 [23-25]
0000746 Cell division variant (Cdi)	19 [6,18]
0000613 Reproductive system physiology variant (Res)	7 [2,4,9,11,15,33,34], 8 [2,4,9,11,15,33,34], 13 [2,4,8,33], 14 [2,4,8,33], 16 [7,21,35], 19 [6,18,36], 26 [21,25,37,38]
No RNAi phenotype recorded in at least two different experiments	1, 2, 3, 4, 5, 9, 11, 12, 20, 21, 23, 24, 25, 27, 28, 29

RNAi phenotypes are based on concordance among different experiments. Only consistent phenotypes described in at least two different experiments were taken into account.

References

1. Kamath RS, Fraser AG, Dong Y, Poulin G, Durbin R, et al. (2003) Systematic functional analysis of the *Caenorhabditis elegans* genome using RNAi. Nature 421: 231-237.

2. Simmer F, Moorman C, van der Linden AM, Kuijk E, van den Berghe PV, et al. (2003) Genome-wide RNAi of *C. elegans* using the hypersensitive *rrf-3* strain reveals novel gene functions. PLoS Biol 1: E12.

3. Kraemer BC, Burgess JK, Chen JH, Thomas JH, Schellenberg GD. (2006) Molecular pathways that influence human tau-induced pathology in *Caenorhabditis elegans*. Hum Mol Genet 15: 1483-1496.

4. Gottschalk A, Almedom RB, Schedletzky T, Anderson SD, Yates JR,3rd, et al. (2005) Identification and characterization of novel nicotinic receptor-associated proteins in *Caenorhabditis elegans*. EMBO J 24: 2566-2578.

5. Anyanful A, Sakube Y, Takuwa K, Kagawa H. (2001) The third and fourth tropomyosin isoforms of *Caenorhabditis elegans* are expressed in the pharynx and intestines and are essential for development and morphology. J Mol Biol 313: 525-537.

6. Skop AR, Liu H, Yates J,3rd, Meyer BJ, Heald R. (2004) Dissection of the mammalian midbody proteome reveals conserved cytokinesis mechanisms. Science 305: 61-66.

7. Ono S, Ono K. (2002) Tropomyosin inhibits ADF/cofilin-dependent actin filament dynamics. J Cell Biol 156: 1065-1076.

8. Cui M, Fay DS, Han M. (2004) Lin-35/Rb cooperates with the SWI/SNF complex to control *Caenorhabditis elegans* larval development. Genetics 167: 1177-1185.

9. Kapulkin WJ, Hiester BG, Link CD. (2005) Compensatory regulation among ER chaperones in *C. elegans*. FEBS Lett 579: 3063-3068.

10. Rual JF, Ceron J, Koreth J, Hao T, Nicot AS, et al. (2004) Toward improving *Caenorhabditis elegans* phenome mapping with an ORFeome-based RNAi library. Genome Res 14: 2162-2168.

11. Sonnichsen B, Koski LB, Walsh A, Marschall P, Neumann B, et al. (2005) Full-genome RNAi profiling of early embryogenesis in *Caenorhabditis elegans*. Nature 434: 462-469.

12. Woo WM, Goncharov A, Jin Y, Chisholm AD. (2004) Intermediate filaments are required for *C. elegans* epidermal elongation. Dev Biol 267: 216-229.

13. Karabinos A, Schmidt H, Harborth J, Schnabel R, Weber K. (2001) Essential roles for four cytoplasmic intermediate filament proteins in *Caenorhabditis elegans* development. Proc Natl Acad Sci U S A 98: 7863-7868.

14. Karabinos A, Schunemann J, Weber K. (2004) Most genes encoding cytoplasmic intermediate filament (IF) proteins of the nematode *Caenorhabditis elegans* are required in late embryogenesis. Eur J Cell Biol 83: 457-468.

15. Piano F, Schetter AJ, Mangone M, Stein L, Kemphues KJ. (2000) RNAi analysis of genes expressed in the ovary of *Caenorhabditis elegans*. Curr Biol 10: 1619-1622.

16. Orsborn AM, Li W, McEwen TJ, Mizuno T, Kuzmin E, et al. (2007) GLH-1, the *C. elegan*s P granule protein, is controlled by the JNK KGB-1 and by the COP9 subunit CSN-5. Development 134: 3383-3392.

17. Yoneda T, Benedetti C, Urano F, Clark SG, Harding HP, et al. (2004) Compartment-specific perturbation of protein handling activates genes encoding mitochondrial chaperones. J Cell Sci 117: 4055-4066.

18. Ai E, Poole DS, Skop AR. (2009) RACK-1 directs dynactin-dependent RAB-11 endosomal recycling during mitosis in *Caenorhabditis elegans*. Mol Biol Cell 20: 1629-1638.

19. Hebeisen M, Drysdale J, Roy R. (2008) Suppressors of the cdc-25.1(gf)-associated intestinal hyperplasia reveal important maternal roles for prp-8 and a subset of splicing factors in *C. elegans*. RNA 14: 2618-2633.

20. Ai E, Poole DS, Skop AR. (2011) Long astral microtubules and RACK-1 stabilize polarity domains during maintenance phase in *Caenorhabditis elegans* embryos. PLoS One 6: e19020.

21. Ceron J, Rual JF, Chandra A, Dupuy D, Vidal M, et al. (2007) Large-scale RNAi screens identify novel genes that interact with the *C. elegans* retinoblastoma pathway as well as splicing-related components with synMuv B activity. BMC Dev Biol 7: 30.

22. Yamashiro S, Gimona M, Ono S. (2007) UNC-87, a calponin-related protein in *C. elegans*, antagonizes ADF/cofilin-mediated actin filament dynamics. J Cell Sci 120: 3022-3033.

23. Heidler T, Hartwig K, Daniel H, Wenzel U. (2010) *Caenorhabditis elegans* lifespan extension caused by treatment with an orally active ROS-generator is dependent on DAF-16 and SIR-2.1. Biogerontology 11: 183-195.

24. Berdichevsky A, Viswanathan M, Horvitz HR, Guarente L. (2006) *C. elegans* SIR-2.1 interacts with 14-3-3 proteins to activate DAF-16 and extend life span. Cell 125: 1165-1177.

25. Li J, Tewari M, Vidal M, Lee SS. (2007) The 14-3-3 protein FTT-2 regulates DAF-16 in *Caenorhabditis elegans*. Dev Biol 301: 82-91.

26. Lamitina T, Huang CG, Strange K. (2006) Genome-wide RNAi screening identifies protein damage as a regulator of osmoprotective gene expression. Proc Natl Acad Sci U S A 103: 12173-12178.

27. Shi A, Sun L, Banerjee R, Tobin M, Zhang Y, et al. (2009) Regulation of endosomal clathrin and retromer-mediated endosome to golgi retrograde transport by the J-domain protein RME-8. EMBO J 28: 3290-3302.

28. Nollen EA, Garcia SM, van Haaften G, Kim S, Chavez A, et al. (2004) Genome-wide RNA interference screen identifies previously undescribed regulators of polyglutamine aggregation. Proc Natl Acad Sci U S A 101: 6403-6408.

29. Haynes CM, Yang Y, Blais SP, Neubert TA, Ron D. (2010) The matrix peptide exporter HAF-1 signals a mitochondrial UPR by activating the transcription factor ZC376.7 in *C. elegans*. Mol Cell 37: 529-540.

30. Balklava Z, Pant S, Fares H, Grant BD. (2007) Genome-wide analysis identifies a general requirement for polarity proteins in endocytic traffic. Nat Cell Biol 9: 1066-1073.

31. Verbrugghe KJ, White JG. (2007) Cortical centralspindlin and G alpha have parallel roles in furrow initiation in early *C. elegans* embryos. J Cell Sci 120: 1772-1778.

32. Ziegler K, Kurz CL, Cypowyj S, Couillault C, Pophillat M, et al. (2009) Antifungal innate immunity in *C. elegans*: PKCdelta links G protein signaling and a conserved p38 MAPK cascade. Cell Host Microbe 5: 341-352.

33. Green RA, Kao HL, Audhya A, Arur S, Mayers JR, et al. (2011) A high-resolution *C. elegans* essential gene network based on phenotypic profiling of a complex tissue. Cell 145: 470-482.

34. Poteryaev D, Squirrell JM, Campbell JM, White JG, Spang A. (2005) Involvement of the actin cytoskeleton and homotypic membrane fusion in ER dynamics in *Caenorhabditis elegans*. Mol Biol Cell 16: 2139-2153.

35. Ono K, Ono S. (2004) Tropomyosin and troponin are required for ovarian contraction in the *Caenorhabditis elegans* reproductive system. Mol Biol Cell 15: 2782-2793.

36. Ciche TA, Sternberg PW. (2007) Postembryonic RNAi in *Heterorhabditis bacteriophora*: A nematode insect parasite and host for insect pathogenic symbionts. BMC Dev Biol 7: 101.

37. Morton DG, Shakes DC, Nugent S, Dichoso D, Wang W, et al. (2002) The *Caenorhabditis elegans* par-5 gene encodes a 14-3-3 protein required for cellular asymmetry in the early embryo. Dev Biol 241: 47-58.

38. Lehner B, Crombie C, Tischler J, Fortunato A, Fraser AG. (2006) Systematic mapping of genetic interactions in *Caenorhabditis elegans* identifies common modifiers of diverse signaling pathways. Nat Genet 38: 896-903.
