# Supplementary material for: Proteomic Analysis of Oesophagostomum dentatum (Nematoda) during Larval Transition, and the Effects of Hydrolase Inhibitors on Development
Source: PLoS One. 2013 May 22;8(5):e63955. doi: 10.1371/journal.pone.0063955 (PMC3661580; doi:10.1371/journal.pone.0063955)
Supplement: Table S3 — A, B. KEGG pathway analysis of the proteins identified. (PDF) [file pone.0063955.s003.pdf]

Table S3A, B. KEGG pathway analysis of the proteins identified.

Table S3A

**BRITE protein classification**	**Number of proteins**
Cellular Processes	
04812 Cytoskeleton proteins [BR:ko04812]	1
Genetic Information Processing	
03009 Ribosome biogenesis [BR:ko03009]	1
03021 Transcription machinery [BR:ko03021]	1
03036 Chromosome [BR:ko03036]	1
03041 Spliceosome [BR:ko03041]	1
03051 Proteasome [BR:ko03051]	1
03110 Chaperones and folding catalysts [BR:ko03110]	3
Metabolism	
01000 Enzymes [BR:ko01000]	7

Table S3B

**Pathway classification**	**Number of proteins**
Cellular Processes	
Cell Communication	1
Cell Growth and Death	1
Cell Motility	1
Transport and Catabolism	3
Environmental Information Processing	
Signal Transduction	2
Genetic Information Processing	
Folding, Sorting and Degradation	3
Transcription	1
Metabolism	
Amino Acid Metabolism	2
Carbohydrate Metabolism	5
Energy Metabolism	2
Unclassified	1
Organismal Systems	
Endocrine System	1
Excretory System	1
Immune System	3
Nervous System	1
Sensory System	1
