# Supplementary material for: Proteomic Analysis of Oesophagostomum dentatum (Nematoda) during Larval Transition, and the Effects of Hydrolase Inhibitors on Development
Source: PLoS One. 2013 May 22;8(5):e63955. doi: 10.1371/journal.pone.0063955 (PMC3661580; doi:10.1371/journal.pone.0063955)
Supplement: Table S4 — Detailed list of the protein assignments to KEGG BRITE protein families and to KEGG biological pathways. (PDF) [file pone.0063955.s004.pdf]

Table S4. Detailed list of the protein assignments to KEGG BRITE protein families and to KEGG biological pathways.

Kterm protein brite annotation

Protein	Kterm	Description	Category 1	Category 2	Category 3
Od_isotig19833_1	K05692	K05692 ACTB_G1; actin beta/gamma 1	Cellular Processes	Cell Motility	04812 Cytoskeleton proteins [BR:ko04812]
Od_isotig20090_1	K05692	K05692 ACTB_G1; actin beta/gamma 1	Cellular Processes	Cell Motility	04812 Cytoskeleton proteins [BR:ko04812]
Od_isotig01423_1	K03283	K03283 HSPA1_8; heat shock 70kDa protein 1/8	Genetic Information Processing	Folding, Sorting and Degradation	03051 Proteasome [BR:ko03051]
Od_isotig01423_1	K03283	K03283 HSPA1_8; heat shock 70kDa protein 1/8	Genetic Information Processing	Folding, Sorting and Degradation	03110 Chaperones and folding catalysts [BR:ko03110]
Od_isotig13569_1	K04077	K04077 groEL, HSPD1; chaperonin GroEL	Genetic Information Processing	Folding, Sorting and Degradation	03110 Chaperones and folding catalysts [BR:ko03110]
Od_isotig17754_1	K08057	K08057 CALR; calreticulin	Genetic Information Processing	Folding, Sorting and Degradation	03110 Chaperones and folding catalysts [BR:ko03110]
Od_isotig19833_1	K05692	K05692 ACTB_G1; actin beta/gamma 1	Genetic Information Processing	Replication and Repair	03036 Chromosome [BR:ko03036]
Od_isotig20090_1	K05692	K05692 ACTB_G1; actin beta/gamma 1	Genetic Information Processing	Replication and Repair	03036 Chromosome [BR:ko03036]
Od_isotig19833_1	K05692	K05692 ACTB_G1; actin beta/gamma 1	Genetic Information Processing	Transcription	03021 Transcription machinery [BR:ko03021]
Od_isotig20090_1	K05692	K05692 ACTB_G1; actin beta/gamma 1	Genetic Information Processing	Transcription	03021 Transcription machinery [BR:ko03021]
Od_isotig01423_1	K03283	K03283 HSPA1_8; heat shock 70kDa protein 1/8	Genetic Information Processing	Transcription	03041 Spliceosome [BR:ko03041]
Od_isotig01423_1	K03283	K03283 HSPA1_8; heat shock 70kDa protein 1/8	Genetic Information Processing	Translation	03009 Ribosome biogenesis [BR:ko03009]
Od_isotig12108_1	K00162	K00162 PDHB, pdhB; pyruvate dehydrogenase E1 component subunit beta [EC:1.2.4.1	Metabolism	Oxidoreductases	01000 Enzymes [BR:ko01000]
Od_isotig07234_1	K00026	K00026 MDH2; malate dehydrogenase [EC:1.1.1.37]	Metabolism	Oxidoreductases	01000 Enzymes [BR:ko01000]
Od_isotig11077_1	K03386	K03386 Peroxiredoxin [EC:1.11.1.15]	Metabolism	Oxidoreductases	01000 Enzymes [BR:ko01000]
Od_isotig22486_1	K01596	K01596 E4.1.1.32, pckA, PEPCK; phosphoenolpyruvate carboxykinase (GTP) [EC:4.1.1	Metabolism	Lyases	01000 Enzymes [BR:ko01000]
Od_isotig23105_1	K01596	K01596 E4.1.1.32, pckA, PEPCK; phosphoenolpyruvate carboxykinase (GTP) [EC:4.1.1	Metabolism	Lyases	01000 Enzymes [BR:ko01000]
Od_isotig21929_1	K01623	K01623 Fructose bisphosphate aldolase, class I [EC:4.1.2.13]	Metabolism	Lyases	01000 Enzymes [BR:ko01000]
Od_isotig21414_1	K01802	K01802 E5.2.1.8; peptidylprolyl isomerase [EC:5.2.1.8]	Metabolism	Isomerases	01000 Enzymes [BR:ko01000]
No contig available	K01965	K01965 Propionyl-CoA carboxylase alpha chain [EC:6.4.1.3]	Metabolism	Ligases	01000 Enzymes [BR:ko01000]

Kterm protein pathway annotation

Protein	Kterm	Kterm description	Category 1	Category 2	Pathway
Od_isotig19833_1	K05692	K05692 ACTB_G1; actin beta/gamma 1	Cellular Processes	Cell Communication	04510 Focal adhesion [PATH:ko04510]
Od_isotig20090_1	K05692	K05692 ACTB_G1; actin beta/gamma 1	Cellular Processes	Cell Communication	04510 Focal adhesion [PATH:ko04510]
Od_isotig19833_1	K05692	K05692 ACTB_G1; actin beta/gamma 1	Cellular Processes	Cell Communication	04520 Adherens junction [PATH:ko04520]
Od_isotig20090_1	K05692	K05692 ACTB_G1; actin beta/gamma 1	Cellular Processes	Cell Communication	04520 Adherens junction [PATH:ko04520]
Od_isotig19833_1	K05692	K05692 ACTB_G1; actin beta/gamma 1	Cellular Processes	Cell Communication	04530 Tight junction [PATH:ko04530]
Od_isotig20090_1	K05692	K05692 ACTB_G1; actin beta/gamma 1	Cellular Processes	Cell Communication	04530 Tight junction [PATH:ko04530]
Od_isotig20385_1	K06630	K06630 YWHA; tyrosine 3-monooxygenase/tryptophan 5-monooxygenase activation pro	Cellular Processes	Cell Growth and Death	04110 Cell cycle [PATH:ko04110]
Od_isotig20385_1	K06630	K06630 YWHA; tyrosine 3-monooxygenase/tryptophan 5-monooxygenase activation pro	Cellular Processes	Cell Growth and Death	04114 Oocyte meiosis [PATH:ko04114]
Od_isotig19833_1	K05692	K05692 ACTB_G1; actin beta/gamma 1	Cellular Processes	Cell Motility	04810 Regulation of actin cytoskeleton [PATH:ko04810]
Od_isotig20090_1	K05692	K05692 ACTB_G1; actin beta/gamma 1	Cellular Processes	Cell Motility	04810 Regulation of actin cytoskeleton [PATH:ko04810]
Od_isotig01423_1	K03283	K03283 HSPA1_8; heat shock 70kDa protein 1/8	Cellular Processes	Transport and Catabolism	04144 Endocytosis [PATH:ko04144]
Od_isotig19833_1	K05692	K05692 ACTB_G1; actin beta/gamma 1	Cellular Processes	Transport and Catabolism	04145 Phagosome [PATH:ko04145]
Od_isotig20090_1	K05692	K05692 ACTB_G1; actin beta/gamma 1	Cellular Processes	Transport and Catabolism	04145 Phagosome [PATH:ko04145]
Od_isotig17754_1	K08057	K08057 CALR; calreticulin	Cellular Processes	Transport and Catabolism	04145 Phagosome [PATH:ko04145]
Od_isotig01423_1	K03283	K03283 HSPA1_8; heat shock 70kDa protein 1/8	Environmental Information Processing	Signal Transduction	04010 MAPK signaling pathway [PATH:ko04010]
Od_isotig14559_1	K15040	K15040 VDAC2; voltage-dependent anion channel protein 2	Environmental Information Processing	Signal Transduction	04020 Calcium signaling pathway [PATH:ko04020]
Od_isotig13569_1	K04077	K04077 groEL, HSPD1; chaperonin GroEL	Genetic Information Processing	Folding, Sorting and Degradation	03018 RNA degradation [PATH:ko03018]
Od_isotig01423_1	K03283	K03283 HSPA1_8; heat shock 70kDa protein 1/8	Genetic Information Processing	Folding, Sorting and Degradation	04141 Protein processing in endoplasmic reticulum [PATH:ko04141]
Od_isotig17754_1	K08057	K08057 CALR; calreticulin	Genetic Information Processing	Folding, Sorting and Degradation	04141 Protein processing in endoplasmic reticulum [PATH:ko04141]
Od_isotig01423_1	K03283	K03283 HSPA1_8; heat shock 70kDa protein 1/8	Genetic Information Processing	Transcription	03040 Spliceosome [PATH:ko03040]
No contig available	K01965	K01965 Propionyl-CoA carboxylase alpha chain [EC:6.4.1.3]	Metabolism	Amino Acid Metabolism	00280 Valine, leucine and isoleucine degradation [PATH:ko00280]
Od_isotig12108_1	K00162	K00162 PDHB, pdhB; pyruvate dehydrogenase E1 component subunit beta [EC:1.2.4.1	Metabolism	Amino Acid Metabolism	00290 Valine, leucine and isoleucine biosynthesis [PATH:ko00290]
Od_isotig12108_1	K00162	K00162 PDHB, pdhB; pyruvate dehydrogenase E1 component subunit beta [EC:1.2.4.1	Metabolism	Carbohydrate Metabolism	00010 Glycolysis / Gluconeogenesis [PATH:ko00010]
Od_isotig22486_1	K01596	K01596 E4.1.1.32, pckA, PEPCK; phosphoenolpyruvate carboxykinase (GTP) [EC:4.1.1	Metabolism	Carbohydrate Metabolism	00010 Glycolysis / Gluconeogenesis [PATH:ko00010]
Od_isotig21929_1	K01623	K01623 Fructose bisphosphate aldolase, class I [EC:4.1.2.13]	Metabolism	Carbohydrate Metabolism	00010 Glycolysis / Gluconeogenesis [PATH:ko00010]
Od_isotig23105_1	K01596	K01596 E4.1.1.32, pckA, PEPCK; phosphoenolpyruvate carboxykinase (GTP) [EC:4.1.1	Metabolism	Carbohydrate Metabolism	00010 Glycolysis / Gluconeogenesis [PATH:ko00010]
Od_isotig07234_1	K00026	K00026 MDH2; malate dehydrogenase [EC:1.1.1.37]	Metabolism	Carbohydrate Metabolism	00020 Citrate cycle (TCA cycle) [PATH:ko00020]
Od_isotig12108_1	K00162	K00162 PDHB, pdhB; pyruvate dehydrogenase E1 component subunit beta [EC:1.2.4.1	Metabolism	Carbohydrate Metabolism	00020 Citrate cycle (TCA cycle) [PATH:ko00020]
Od_isotig22486_1	K01596	K01596 E4.1.1.32, pckA, PEPCK; phosphoenolpyruvate carboxykinase (GTP) [EC:4.1.1	Metabolism	Carbohydrate Metabolism	00020 Citrate cycle (TCA cycle) [PATH:ko00020]
Od_isotig23105_1	K01596	K01596 E4.1.1.32, pckA, PEPCK; phosphoenolpyruvate carboxykinase (GTP) [EC:4.1.1	Metabolism	Carbohydrate Metabolism	00020 Citrate cycle (TCA cycle) [PATH:ko00020]
Od_isotig21929_1	K01623	K01623 Fructose bisphosphate aldolase, class I [EC:4.1.2.13]	Metabolism	Carbohydrate Metabolism	00030 Pentose phosphate pathway [PATH:ko00030]
Od_isotig21929_1	K01623	K01623 Fructose bisphosphate aldolase, class I [EC:4.1.2.13]	Metabolism	Carbohydrate Metabolism	00051 Fructose and mannose metabolism [PATH:ko00051]
Od_isotig07234_1	K00026	K00026 MDH2; malate dehydrogenase [EC:1.1.1.37]	Metabolism	Carbohydrate Metabolism	00620 Pyruvate metabolism [PATH:ko00620]
Od_isotig12108_1	K00162	K00162 PDHB, pdhB; pyruvate dehydrogenase E1 component subunit beta [EC:1.2.4.1	Metabolism	Carbohydrate Metabolism	00620 Pyruvate metabolism [PATH:ko00620]
Od_isotig22486_1	K01596	K01596 E4.1.1.32, pckA, PEPCK; phosphoenolpyruvate carboxykinase (GTP) [EC:4.1.1	Metabolism	Carbohydrate Metabolism	00620 Pyruvate metabolism [PATH:ko00620]
Od_isotig23105_1	K01596	K01596 E4.1.1.32, pckA, PEPCK; phosphoenolpyruvate carboxykinase (GTP) [EC:4.1.1	Metabolism	Carbohydrate Metabolism	00620 Pyruvate metabolism [PATH:ko00620]
Od_isotig07234_1	K00026	K00026 MDH2; malate dehydrogenase [EC:1.1.1.37]	Metabolism	Carbohydrate Metabolism	00630 Glyoxylate and dicarboxylate metabolism [PATH:ko00630]
No contig available	K01965	K01965 Propionyl-CoA carboxylase alpha chain [EC:6.4.1.3]	Metabolism	Carbohydrate Metabolism	00630 Glyoxylate and dicarboxylate metabolism [PATH:ko00630]
No contig available	K01965	K01965 Propionyl-CoA carboxylase alpha chain [EC:6.4.1.3]	Metabolism	Carbohydrate Metabolism	00640 Propanoate metabolism [PATH:ko00640]
Od_isotig12108_1	K00162	K00162 PDHB, pdhB; pyruvate dehydrogenase E1 component subunit beta [EC:1.2.4.1	Metabolism	Carbohydrate Metabolism	00650 Butanoate metabolism [PATH:ko00650]

Od_isotig07234_1	K00026	K00026 MDH2; malate dehydrogenase [EC:1.1.1.37]	Metabolism	Energy Metabolism	00710 Carbon fixation in photosynthetic organisms [PATH:ko00710]
Od_isotig21929_1	K01623	K01623 Fructose bisphosphate aldolase, class I [EC:4.1.2.13]	Metabolism	Energy Metabolism	00710 Carbon fixation in photosynthetic organisms [PATH:ko00710]
Od_isotig22486_1	K01596	K01596 E4.1.1.32, pckA, PEPCK; phosphoenolpyruvate carboxykinase (GTP) [EC:4.1.1	Organismal Systems	Endocrine System	03320 PPAR signaling pathway [PATH:ko03320]
Od_isotig23105_1	K01596	K01596 E4.1.1.32, pckA, PEPCK; phosphoenolpyruvate carboxykinase (GTP) [EC:4.1.1	Organismal Systems	Endocrine System	03320 PPAR signaling pathway [PATH:ko03320]
Od_isotig22486_1	K01596	K01596 E4.1.1.32, pckA, PEPCK; phosphoenolpyruvate carboxykinase (GTP) [EC:4.1.1	Organismal Systems	Endocrine System	04910 Insulin signaling pathway [PATH:ko04910]
Od_isotig23105_1	K01596	K01596 E4.1.1.32, pckA, PEPCK; phosphoenolpyruvate carboxykinase (GTP) [EC:4.1.1	Organismal Systems	Endocrine System	04910 Insulin signaling pathway [PATH:ko04910]
Od_isotig22486_1	K01596	K01596 E4.1.1.32, pckA, PEPCK; phosphoenolpyruvate carboxykinase (GTP) [EC:4.1.1	Organismal Systems	Endocrine System	04920 Adipocytokine signaling pathway [PATH:ko04920]
Od_isotig23105_1	K01596	K01596 E4.1.1.32, pckA, PEPCK; phosphoenolpyruvate carboxykinase (GTP) [EC:4.1.1	Organismal Systems	Endocrine System	04920 Adipocytokine signaling pathway [PATH:ko04920]
Od_isotig22486_1	K01596	K01596 E4.1.1.32, pckA, PEPCK; phosphoenolpyruvate carboxykinase (GTP) [EC:4.1.1	Organismal Systems	Excretory System	04964 Proximal tubule bicarbonate reclamation [PATH:ko04964]
Od_isotig23105_1	K01596	K01596 E4.1.1.32, pckA, PEPCK; phosphoenolpyruvate carboxykinase (GTP) [EC:4.1.1	Organismal Systems	Excretory System	04964 Proximal tubule bicarbonate reclamation [PATH:ko04964]
Od_isotig01423_1	K03283	K03283 HSPA1_8; heat shock 70kDa protein 1/8	Organismal Systems	Immune System	04612 Antigen processing and presentation [PATH:ko04612]
Od_isotig17754_1	K08057	K08057 CALR; calreticulin	Organismal Systems	Immune System	04612 Antigen processing and presentation [PATH:ko04612]
Od_isotig19833_1	K05692	K05692 ACTB_G1; actin beta/gamma 1	Organismal Systems	Immune System	04670 Leukocyte transendothelial migration [PATH:ko04670]
Od_isotig20090_1	K05692	K05692 ACTB_G1; actin beta/gamma 1	Organismal Systems	Immune System	04670 Leukocyte transendothelial migration [PATH:ko04670]
Od_isotig20385_1	K06630	K06630 YWHA; tyrosine 3-monooxygenase/tryptophan 5-monooxygenase activation pro	Organismal Systems	Nervous System	04722 Neurotrophin signaling pathway [PATH:ko04722]
Od_isotig19833_1	K05692	K05692 ACTB_G1; actin beta/gamma 1	Organismal Systems	Sensory System	04745 Phototransduction - fly [PATH:ko04745]
Od_isotig20090_1	K05692	K05692 ACTB_G1; actin beta/gamma 1	Organismal Systems	Sensory System	04745 Phototransduction - fly [PATH:ko04745]
Od_isotig11077_1	K03386	K03386 Peroxiredoxin [EC:1.11.1.15]	unclassified		
