# Supplementary material for: Proteomic Analysis of Oesophagostomum dentatum (Nematoda) during Larval Transition, and the Effects of Hydrolase Inhibitors on Development
Source: PLoS One. 2013 May 22;8(5):e63955. doi: 10.1371/journal.pone.0063955 (PMC3661580; doi:10.1371/journal.pone.0063955)
Supplement: Table S5 — A, B. Gene Ontology (GO) analysis of the proteins identified. (PDF) [file pone.0063955.s005.pdf]

Table S5A, B. Gene Ontology (GO) analysis of the proteins identified.

Table S5A

**GO classification - Biological process**	**Number of proteins**
GO:0000003 Reproduction	8
GO:0022414 Reproductive process	5
GO:0008152 Metabolic process	10
GO:0006807 Nitrogen compound metabolic process	1
GO:0009056 Catabolic process	1
GO:0009058 Biosynthetic process	1
GO:0019637 Organophosphate metabolic process	1
GO:0044237 Cellular metabolic process	7
GO:0044238 Primary metabolic process	2
GO:0044281 Small molecule metabolic process	2
GO:0009987 Cellular process	9
GO:0000910 Cytokinesis	1
GO:0006928 Cellular component movement	1
GO:0007049 Cell cycle	1
GO:0007059 Chromosome segregation	1
GO:0008219 Cell death	1
GO:0048610 Cellular process involved in reproduction	1
GO:0048869 Cellular developmental process	1
GO:0051301 Cell division	1
GO:0051641 Cellular localization	1
GO:0051716 Cellular response to stimulus	1
GO:0071841 Cellular component organization or biogenesis at cellular level	5
GO:0032501 Multicellular organismal process	11
GO:0003008 System process	1
GO:0007275 Multicellular organismal development	11
GO:0032504 Multicellular organism reproduction	4
GO:0042303 Molting cycle	2
GO:0051239 Regulation of multicellular organismal process	1
GO:0032502 Developmental process	11
GO:0007568 Aging	2
GO:0009791 Post-embryonic development	9
GO:0048856 Anatomical structure development	10
GO:0040007 Growth	11
GO:0040008 Regulation of growth	1
GO:0040011 Locomotion	8
GO:0050896 Response to stimulus	8
GO:0006950 Response to stress	4
GO:0051179 Localization	4
GO:0051234 Establishment of localization	3
GO:0051641 Cellular localization	1
GO:0051674 Localization of cell	1
GO:0065007 Biological regulation	8
GO:0050789 Regulation of biological process	8
GO:0065009 Regulation of molecular function	1
No biological process described	3

Only annotations in related nematode species were taken into account with a cut-off of $p \leq 2*10^{-25}$.

Table S5B

**GO classification - Molecular function**	**Number of proteins**
GO:0003824 Catalytic activity	10
GO:0016491 Oxireductase activity	2
GO:0016787 Hydrolase activity	2
GO:0016829 Lyase activity	2
GO:0016853 Isomerase activity	1
GO:0016874 Ligase activity	1
GO:0005198 Structural molecule activity	2
GO:0005215 Transporter activity	1
GO:0022857 Transmembrane transporter activity	1
GO:0022892 Substrate-specific transporter activity	1
GO:0005488 Binding	11
GO:0005515 Protein binding	7
GO:0036094 Small molecule binding	4
GO:0043167 Ion binding	3
GO:0097159 Organic cyclic compound binding	4
GO:0016209 Antioxidant activity	1
GO:0004601 Peroxidase activity	1
No molecular function described	4

Only annotations in related nematode species were taken into account with a cut-off of $p \leq 2*10^{-25}$.
