# Supplementary material for: Proteomic Analysis of Oesophagostomum dentatum (Nematoda) during Larval Transition, and the Effects of Hydrolase Inhibitors on Development
Source: PLoS One. 2013 May 22;8(5):e63955. doi: 10.1371/journal.pone.0063955 (PMC3661580; doi:10.1371/journal.pone.0063955)
Supplement: Table S6 — Gene Ontology (GO) assigments of proteins identified. The conceptually translated amino acid sequences of the identified Oesophagostomum dentatum contigs were applied to GO analyses using the AmiGO tool. Only annotations found in related nematode species were taken into account using a cut-off of p≤2×10^−25^. (PDF) [file pone.0063955.s006.pdf]

Table S6. Gene Ontology (GO) assigments of proteins identified. The conceptually translated amino acid sequences of the identified *Oesophagostomum dentatum* contigs were applied to GO analyses using the AmiGO tool. Only annotations found in related nematode species were taken into account using a cut-off of $p \leq 2x10^{-25}$.

GO - Biological process

Protein	Category 1	Category 2	Category 3
LIM domain protein	GO:0000003 Reproduction		
Propionyl-CoA carboxylase alpha chain	GO:0008152 Metabolic process		
Phosphoenolpyruvate carboxy kinase GTP	GO:0008152 Metabolic process	GO:0044238 Primary metabolic process	GO:0006094 Gluconeogenesis
		GO:0009058 Biosynthetic process	
Intermediate filament protein B	GO:0032501 Multicellular organismal process	GO:0007275 Multicellular organismal development	GO:0002119 Nematode larval development
	GO:0032502 Developmental process	GO:0009791 Post-embryonic development	
	GO:0032502 Developmental process	GO:0007568 Aging	GO:0008340 Determination of adult lifespan
	GO:0032501 Multicellular organismal process	GO:0007275 Multicellular organismal development	
	GO:0032502 Developmental process	GO:0048856 Anatomical structure development	GO:0009792 Embryo development ending in birth or egg hatching
	GO:0032501 Multicellular organismal process	GO:0007275 Multicellular organismal development	
	GO:0032502 Developmental process	GO:0048856 Anatomical structure development	GO:0010171 Body morphogenesis
	GO:0032501 Multicellular organismal process	GO:0042303 Molting cycle	GO:0018996 Molting cycle, collagen and cuticulin-based cuticle
	GO:0032502 Developmental process	GO:0048856 Anatomical structure development	GO:0040002 Collagen and cuticulin-based cuticle development
	GO:0032501 Multicellular organismal process	GO:0007275 Multicellular organismal development	
	GO:0040007 Growth		
	GO:0040011 Locomotion		
Heat shock 70 kDa protein	GO:0000003 Reproduction		
	GO:0032501 Multicellular organismal process	GO:0007275 Multicellular organismal development	GO:0002119 Nematode larval development
	GO:0032502 Developmental process	GO:0009791 Post-embryonic development	
	GO:0008152 Metabolic process	GO:0044237 Cellular metabolic process	GO:0006200 ATP catabolic process
		GO:0044281 Small molecule metabolic process	
		GO:0019637 Organophosphate metabolic process	
		GO:0006807 Nitrogen compound metabolic process	
		GO:0009056 Catabolic process	
	GO:0009987 Cellular process	GO:0044237 Cellular metabolic process	
	GO:0032502 Developmental process	GO:0007568 Aging	GO:0008340 Determination of adult lifespan
	GO:0032501 Multicellular organismal process	GO:0007275 Multicellular organismal development	
	GO:0050896 Response to stimulus	GO:0006950 Response to stress	GO:0009408 Response to heat
	GO:0032502 Developmental process	GO:0048856 Anatomical structure development	GO:0009792 Embryo development ending in birth or egg hatching
	GO:0032501 Multicellular organismal process	GO:0007275 Multicellular organismal development	
	GO:0032502 Developmental process	GO:0048856 Anatomical structure development	GO:0010171 Body morphogenesis
	GO:0040007 Growth		
	GO:0040011 Locomotion		
	GO:0009987 Cellular process	GO:0051641 Cellular localization	GO:0042147 Retrograde transport, endosome to Golgi
	GO:0051179 Localization	GO:0051234 Establishment of localization	
Troponin T	GO:0065007 Biological regulation	GO:0050789 Regulation of biological process	GO:0040010 Positive regulation of growth rate
	GO:0040007 Growth		
	GO:0040011 Locomotion		
	GO:0040011 Locomotion		GO:0040017 Positive regulation of locomotion
Heat shock 60 kDa protein	GO:0000003 Reproduction		
	GO:0050896 Response to stimulus	GO:0006950 Response to stress	GO:0000302 Response to reactive oxygen species
	GO:0032501 Multicellular organismal process	GO:0007275 Multicellular organismal development	GO:0002119 Nematode larval development
	GO:0032502 Developmental process	GO:0009791 Post-embryonic development	
	GO:0008152 Metabolic process	GO:0044237 Cellular metabolic process	GO:0006457 Protein folding
	GO:0009987 Cellular process		
	GO:0009987 Cellular process	GO:0071841 Cellular component organization or biogenesis at cellular level	GO:0007005 Mitochondrion organization
	GO:0050896 Response to stimulus	GO:0006950 Response to stress	GO:0009408 Response to heat
	GO:0032502 Developmental process	GO:0048856 Anatomical structure development	GO:0009792 Embryo development ending in birth or egg hatching
	GO:0032501 Multicellular organismal process	GO:0007275 Multicellular organismal development	
	GO:0050896 Response to stimulus	GO:0006950 Response to stress	GO:0034514 Mitochondrial unfolded protein response
	GO:0009987 Cellular process	GO:0051716 Cellular response to stimulus	
	GO:0040007 Growth		
	GO:0065007 Biological regulation	GO:0050789 Regulation of biological process	GO:0040010 Positive regulation of growth rate
	GO:0040007 Growth		
	GO:0008152 Metabolic process	GO:0044237 Cellular metabolic process	GO:0042026 Protein refolding
	GO:0009987 Cellular process		

	GO:0008152 Metabolic process	GO:0044237 Cellular metabolic process	GO:0044267 Cellular protein metabolic process
	GO:0009987 Cellular process		
4-hydroxybutyrate coenzyme A transferase	GO:0008152 Metabolic process	GO:0044237 Cellular metabolic process	GO:0006084 Acetyl-CoA metabolic process
	GO:0009987 Cellular process		
Calreticulin	GO:0032501 Multicellular organismal process	GO:0007275 Multicellular organismal development	GO:0002119 Nematode larval development
	GO:0032502 Developmental process	GO:0009791 Post-embryonic development	
	GO:0008152 Metabolic process	GO:0044237 Cellular metabolic process	GO:0006457 Protein folding
	GO:0009987 Cellular process		
	GO:0050896 Response to stimulus		GO:0006950 Response to stress
	GO:0009987 Cellular process	GO:0008219 Cell death	GO:0012501 Programmed cell death
	GO:0016265 Death	GO:0008219 Cell death	
	GO:0032501 Multicellular organismal process	GO:0003008 System process	GO:0030421 Defecation
	GO:0009987 Cellular process	GO:0071841 Cellular component organization or biogenesis at cellular level	GO:0031581 Hemidesmosome assembly
	GO:0040011 Locomotion		
	GO:0032501 Multicellular organismal process		GO:0048609 Multicellular organismal reproductive process
Actin	GO:0000003 Reproduction		
	GO:0009987 Cellular process		GO:0000910 Cytokinesis
	GO:0032501 Multicellular organismal process	GO:0007275 Multicellular organismal development	GO:0002119 Nematode larval development
	GO:0032502 Developmental process	GO:0009791 Post-embryonic development	
	GO:0032502 Developmental process	GO:0048856 Anatomical structure development	GO:0009792 Embryo development ending in birth or egg hatching
	GO:0032501 Multicellular organismal process	GO:0007275 Multicellular organismal development	
	GO:0009987 Cellular process	GO:0071841 Cellular component organization or biogenesis at cellular level	GO:0030036 Actin cytoskeleton organization
	GO:0040007 Growth		
	GO:0040011 Locomotion		
	GO:0032502 Developmental process	GO:0048856 Anatomical structure development	GO:0040035 Hermaphrodite genitalia development
	GO:0032501 Multicellular organismal process	GO:0007275 Multicellular organismal development	
	GO:0000003 Reproduction	GO:0022414 Reproductive process	
Fructose-bisphosphate aldolase	GO:0000003 Reproduction		
	GO:0008152 Metabolic process	GO:0044237 Cellular metabolic process	GO:0006096 Glycolysis
	GO:0009987 Cellular process		
	GO:0032502 Developmental process	GO:0048856 Anatomical structure development	GO:0009792 Embryo development ending in birth or egg hatching
	GO:0032501 Multicellular organismal process	GO:0007275 Multicellular organismal development	
	GO:0032501 Multicellular organismal process	GO:0032504 Multicellular organism reproduction	GO:0018991 Oviposition
	GO:0000003 Reproduction	GO:0022414 Reproductive process	
	GO:0050896 Response to stimulus	GO:0007610 Behavior	
	GO:0065007 Biological regulation	GO:0050789 Regulation of biological process	GO:0040010 Positive regulation of growth rate
	GO:0040007 Growth		
	GO:0040011 Locomotion		
Tropomyosin	GO:0000003 Reproduction		
	GO:0009987 Cellular process		GO:0000910 Cytokinesis
	GO:0032501 Multicellular organismal process	GO:0007275 Multicellular organismal development	GO:0002119 Nematode larval development
	GO:0032502 Developmental process	GO:0009791 Post-embryonic development	
	GO:0032502 Developmental process	GO:0048856 Anatomical structure development	GO:0009792 Embryo development ending in birth or egg hatching
	GO:0032501 Multicellular organismal process	GO:0007275 Multicellular organismal development	
	GO:0032502 Developmental process	GO:0048856 Anatomical structure development	GO:0010171 Body morphogenesis
	GO:0032501 Multicellular organismal process	GO:0032504 Multicellular organism reproduction	GO:0018991 Oviposition
	GO:0000003 Reproduction	GO:0022414 Reproductive process	
	GO:0050896 Response to stimulus	GO:0007610 Behavior	
	GO:0032501 Multicellular organismal process	GO:0042303 Molting cycle	GO:0018996 Molting cycle, collagen and cuticulin-based cuticle
	GO:0009987 Cellular process	GO:0071841 Cellular component organization or biogenesis at cellular level	GO:0030833 Regulation of actin filament depolymerization
	GO:0065007 Biological regulation	GO:0050789 Regulation of biological process	
	GO:0065007 Biological regulation	GO:0050789 Regulation of biological process	GO:0030835 Negative regulation of actin filament depolymerization
	GO:0040011 Locomotion		
	GO:0040011 Locomotion		GO:0040017 Positive regulation of locomotion
	GO:0065007 Biological regulation	GO:0050789 Regulation of biological process	GO:0040015 Negative regulation of multicellular organism growth
	GO:0040007 Growth	GO:0040008 Regulation of growth	
	GO:0032501 Multicellular organismal process	GO:0051239 Regulation of multicellular organismal process	
	GO:0032502 Developmental process	GO:0048856 Anatomical structure development	GO:0040035 Hermaphrodite genitalia development
	GO:0032501 Multicellular organismal process	GO:0007275 Multicellular organismal development	
	GO:0000003 Reproduction	GO:0022414 Reproductive process	
	GO:0065007 Biological regulation	GO:0065009 Regulation of molecular function	GO:0043393 Regulation of protein binding

Malate dehydrogenase	GO:0032501 Multicellular organismal process	GO:0007275 Multicellular organismal development	GO:0002119 Nematode larval development
	GO:0032502 Developmental process	GO:0009791 Post-embryonic development	
	GO:0008152 Metabolic process	GO:0044237 Cellular metabolic process	GO:0006108 Malate metabolic process
		GO:0044281 Small molecule metabolic process	
	GO:0009987 Cellular process		
	GO:0032502 Developmental process	GO:0048856 Anatomical structure development	GO:0009792 Embryo development ending in birth or egg hatching
	GO:0032501 Multicellular organismal process	GO:0007275 Multicellular organismal development	
	GO:0040007 Growth		
	GO:0065007 Biological regulation	GO:0050789 Regulation of biological process	GO:0040010 Positive regulation of growth rate
	GO:0040007 Growth		
	GO:0008152 Metabolic process	GO:0044238 Primary metabolic process	GO:0044262 Cellular carbohydrate metabolic process
	GO:0009987 Cellular process	GO:0044237 Cellular metabolic process	
	GO:0008152 Metabolic process		GO:0055114 Oxidation-reduction process
RACK-1 (receptor for activated protein kinase C 1)	GO:0009987 Cellular process	GO:0007049 Cell cycle	GO:0000281 Cytokinesis after mitosis
		GO:0051301 Cell division	
		GO:0000910 Cytokinesis	
	GO:0032501 Multicellular organismal process	GO:0007275 Multicellular organismal development	GO:0002119 Nematode larval development
	GO:0032502 Developmental process	GO:0009791 Post-embryonic development	
	GO:0009987 Cellular process	GO:0071841 Cellular component organization or biogenesis at cellular level	GO:0006997 Nucleus organization
	GO:0032501 Multicellular organismal process	GO:0007275 Multicellular organismal development	GO:0008045 Motor axon guidance
	GO:0009987 Cellular process	GO:0048869 Cellular developmental process	
	GO:0032502 Developmental process	GO:0048869 Cellular developmental process	
	GO:0032502 Developmental process	GO:0048856 Anatomical structure development	GO:0009792 Embryo development ending in birth or egg hatching
	GO:0032501 Multicellular organismal process	GO:0007275 Multicellular organismal development	
	GO:0032501 Multicellular organismal process	GO:0032504 Multicellular organism reproduction	GO:0018991 Oviposition
	GO:0000003 Reproduction	GO:0022414 Reproductive process	
	GO:0050896 Response to stimulus	GO:0007610 Behavior	
	GO:0065007 Biological regulation	GO:0050789 Regulation of biological process	GO:0030334 Regulation of cell migration
	GO:0009987 Cellular process	GO:0006928 Cellular component movement	
	GO:0051179 Localization		
	GO:0009987 Cellular process	GO:0051641 Cellular localization	GO:0033365 Protein localization to organelle
	GO:0051179 Localization	GO:0051641 Cellular localization	
	GO:0040007 Growth		
	GO:0065007 Biological regulation	GO:0050789 Regulation of biological process	GO:0040010 Positive regulation of growth rate
	GO:0040007 Growth		
	GO:0040011 Locomotion		GO:0040017 Positive regulation if locomotion
	GO:0032502 Developmental process	GO:0048856 Anatomical structure development	GO:0040035 Hermaphrodite genitalia development
	GO:0032501 Multicellular organismal process	GO:0007275 Multicellular organismal development	
	GO:0000003 Reproduction	GO:0022414 Reproductive process	
	GO:0000003 Reproduction	GO:0022414 Reproductive process	GO:0040038 Polar body extrusion after meiotic division
	GO:0009987 Cellular process	GO:0048610 Cellular process involved in reproduction	
	GO:0009987 Cellular process	GO:0007049 Cell cycle	GO:0051304 Chromosome separation
		GO:0007059 Chromosome segregation	
Disorganised muscle protein 1	No GO data reported		No gene ontology data
Pyruvate Dehydrogenase E1	GO:0008152 Metabolic process		
	GO:0032502 Developmental process	GO:0048856 Anatomical structure development	GO:0009792 Embryo development ending in birth or egg hatching
	GO:0032501 Multicellular organismal process	GO:0007275 Multicellular organismal development	
	GO:0065007 Biological regulation	GO:0050789 Regulation of biological process	GO:0040010 Positive regulation of growth rate
	GO:0040007 Growth		
Probable voltage-dependent anion-selective channel	GO:0051179 Localization	GO:0051234 Establishment of localization	GO:0006820 Anion transport
	GO:0051179 Localization	GO:0051234 Establishment of localization	GO:0044070 Regulation of anion transport
	GO:0051179 Localization	GO:0051234 Establishment of localization	GO:0055085 Transmembrane transport
Aspartyl protease inhibitor	No GO data reported		No gene ontology data
14-3-3 protein	GO:0032501 Multicellular organismal process	GO:0007275 Multicellular organismal development	GO:0002119 Nematode larval development
	GO:0032502 Developmental process	GO:0009791 Post-embryonic development	
	GO:0032502 Developmental process	GO:0048856 Anatomical structure development	GO:0009792 Embryo development ending in birth or egg hatching
	GO:0032501 Multicellular organismal process	GO:0007275 Multicellular organismal development	
	GO:0032501 Multicellular organismal process	GO:0032504 Multicellular organism reproduction	GO:0018991 Oviposition
	GO:0000003 Reproduction	GO:0022414 Reproductive process	
	GO:0050896 Response to stimulus	GO:0007610 Behavior	
	GO:0040007 Growth		

	GO:0065007 Biological regulation	GO:0050789 Regulation of biological process	GO:0040010 Positive regulation of growth rate
	GO:0040007 Growth		
	GO:0032501 Multicellular organismal process	GO:0007275 Multicellular organismal development	GO:0043053 Dauer entry
	GO:0032502 Developmental process	GO:0009791 Post-embryonic development	
Phosphatidylethanol-amine binding protein homolog	No GO data reported		No gene ontology data
Peroxiredoxin	GO:0050896 Response to stimulus	GO:0006950 Response to stress	GO:0006979 Response to oxidative stress
Peptidyl-prolyl *cis-trans* isomerase	GO:0008152 Metabolic process	GO:0044237 Cellular metabolic process	GO:0000413 Protein peptidyl-prolyl isomerization
	GO:0009987 Cellular process		
	GO:0008152 Metabolic process	GO:0044237 Cellular metabolic process	GO:0006457 Protein folding
	GO:0009987 Cellular process		

GO - Molecular function

Protein	Category 1	Category 2	Category3
LIM domain protein	GO:0005488 Binding	GO:0043167 Ion binding	GO:0008270 Zinc ion binding
Propionyl-CoA carboxylase alpha chain	GO:0003824 Catalytic activity		
	GO:0005488 Binding	GO:0036094 Small molecule binding	GO:0005524 ATP binding
		GO:0097159 Organic cyclic compound binding	
	GO:0003824 Catalytic activity	GO:0016874 Ligase activity	GO:0008716 D-alanine-D-alanine ligase activity
	GO:0003824 Catalytic activity		GO:0016874 Ligase activity
	GO:0005488 Binding	GO:0043167 Ion binding	GO:0046872 Metal ion binding
Phosphoenolpyruvate carboxy kinase GTP	GO:0003824 Catalytic activity	GO:0016829 Lyase activity	GO:0004611 Phosphoenolpyruvate carboxy kinase activity
	GO:0003824 Catalytic activity	GO:0016829 Lyase activity	GO:0004613 Phosphoenolpyruvate carboxy kinase (GTP) activity
	GO:0005488 Binding	GO:0036094 Small molecule binding	GO:0005525 GTP binding
		GO:0097159 Organic cyclic compound binding	
Intermediate filament protein B	GO:0005198 Structural molecule activity		
	GO:0005488 Binding		GO:0005515 Protein binding
Heat shock 70 kDa protein	GO:0005488 Binding		GO:0005515 Protein binding
	GO:0005488 Binding	GO:0036094 Small molecule binding	GO:0005524 ATP-binding
		GO:0097159 Organic cyclic compound binding	
	GO:0003824 Catalytic activity	GO:0016787 Hydrolase activity	GO:0016887 ATPase activity
Troponin T	No GO data reported		No gene ontology data
Heat shock 60 kDa protein	GO:0005488 Binding	GO:0005515 Protein binding	GO:0008134 Transcription factor binding
	GO:0003824 Catalytic activity	GO:0016787 Hydrolase activity	GO:0016887 ATPase activity
	GO:0005488 Binding	GO:0005515 Protein binding	GO:0051082 Unfolded protein binding
4-hydroxybutyrate coenzyme A transferase	GO:0003824 Catalytic activity		
Calreticulin	GO:0005488 Binding	GO:0043167 Ion binding	GO:0005509 Calcium ion binding
	GO:0005488 Binding	GO:0005515 Protein binding	GO:0051082 Unfolded protein binding
Actin	GO:0005198 Structural molecule activity		GO:0005200 Structural constituent of exoskeleton
	GO:0005488 Binding	GO:0036094 Small molecule binding	GO:0005524 ATP-binding
		GO:0097159 Organic cyclic compound binding	
Fructose-bisphosphate aldolase	GO:0003824 Catalytic activity	GO:0016829 Lyase activity	GO:0004332 Fructose-biphosphate aldolase activity
Tropomyosin	GO:0005488 Binding	GO:0005515 Protein binding	GO:0051015 Actin filament binding
Malate dehydrogenase	GO:0003824 Catalytic activity		GO:0016491 Oxireductase activity
	GO:0003824 Catalytic activity	GO:0016491 Oxireductase activity	GO:0016615 Malate dehydrogenase activity
	GO:0003824 Catalytic activity	GO:0016491 Oxireductase activity	GO:0030060 L-malate dehydrogenase activity
RACK-1 (receptor for activated protein kinase C 1)	GO:0005488 Binding		GO:0005515 Protein binding
Disorganised muscle protein 1	No GO data reported		
Pyruvate Dehydrogenase E1	GO:0003824 Catalytic activity		
Probable voltage-dependent anion-selective channel	GO:0005215 Transporter activity	GO:0022857 Transmembrane transporter activity	GO:0008308 Voltage-gated anion channel activity
		GO:0022892 Substrate-specific transporter activity	
Aspartyl protease inhibitor	No GO data reported		No gene ontology data
14-3-3 protein	GO:0005488 Binding		GO:0005515 Protein binding
	GO:0005488 Binding	GO:0005515 Protein binding	GO:0019904 Protein domain specific binding
Phosphatidylethanol-amine binding protein homolog	No GO data reported		
Peroxiredoxin	GO:0016209 Antioxidant activity		GO:0004601 Peroxidase activity
	GO:0003824 Catalytic activity	GO:0016491 Oxireductase activity	
	GO:0016209 Antioxidant activity	GO:0004601 Peroxidase activity	GO:0008379 Thioredoxin peroxidase activity
	GO:0003824 Catalytic activity	GO:0016491 Oxireductase activity	
	GO:0016209 Antioxidant activity		
Peptidyl-prolyl *cis-trans* isomerase	GO:0003824 Catalytic activity	GO:0016853 Isomerase activity	GO:0003755 Protin peptidyl-prolyl isomerase activity
